# AtOMA1 Affects the OXPHOS System and Plant Growth in Contrast to Other Newly Identified ATP-Independent Proteases in Arabidopsis Mitochondria

**DOI:** 10.3389/fpls.2017.01543

**Published:** 2017-09-07

**Authors:** Iwona Migdal, Renata Skibior-Blaszczyk, Malgorzata Heidorn-Czarna, Marta Kolodziejczak, Arnold Garbiec, Hanna Janska

**Affiliations:** ^1^Institute of Experimental Biology, Faculty of Biological Sciences, University of Wroclaw Wroclaw, Poland; ^2^Department of Cellular Molecular Biology, Faculty of Biotechnology, University of Wroclaw Wroclaw, Poland

**Keywords:** ATP-independent proteases, OMA1, plant mitochondria, OXPHOS, complex V, *Arabidopsis thaliana*

## Abstract

Compared with yeast, our knowledge on members of the ATP-independent plant mitochondrial proteolytic machinery is rather poor. In the present study, using confocal microscopy and immunoblotting, we proved that homologs of yeast Oma1, Atp23, Imp1, Imp2, and Oct1 proteases are localized in Arabidopsis mitochondria. We characterized these components of the ATP-independent proteolytic system as well as the earlier identified protease, AtICP55, with an emphasis on their significance in plant growth and functionality in the OXPHOS system. A functional complementation assay demonstrated that out of all the analyzed proteases, only AtOMA1 and AtICP55 could substitute for a lack of their yeast counterparts. We did not observe any significant developmental or morphological changes in plants lacking the studied proteases, either under optimal growth conditions or after exposure to stress, with the only exception being retarded root growth in *oma1-1*, thus implying that the absence of a single mitochondrial ATP-independent protease is not critical for Arabidopsis growth and development. We did not find any evidence indicating a clear functional complementation of the missing protease by any other protease at the transcript or protein level. Studies on the impact of the analyzed proteases on mitochondrial bioenergetic function revealed that out of all the studied mutants, only *oma1-1* showed differences in activities and amounts of OXPHOS proteins. Among all the OXPHOS disorders found in *oma1-1*, the complex V deficiency is distinctive because it is mainly associated with decreased catalytic activity and not correlated with complex abundance, which has been observed in the case of supercomplex I + III_2_ and complex I deficiencies. Altogether, our study indicates that despite the presence of highly conservative homologs, the mitochondrial ATP-independent proteolytic system is not functionally conserved in plants as compared with yeast. Our findings also highlight the importance of AtOMA1 in maintenance of proper function of the OXPHOS system as well as in growth and development of *Arabidopsis thaliana*.

## Introduction

The proper functioning of mitochondria requires an efficient system for the maintenance of quantity and quality of the mitochondrial proteome. Besides chaperones, the key players in this system are mitoproteases, which can be classified into two groups based on an ATP requirement for their activity. ATP-dependent proteases are involved in degradation of misfolded or damaged proteins. Being processing peptidases, these are also required for native regulatory protein turnover (Janska et al., 2010; Quirós et al., 2015; van Wijk, 2015). On the other hand, ATP-independent proteases are largely involved in limited proteolytic cleavage of presequences and protein maturation. However, this type of proteases can also degrade proteins, e.g., Deg (degradation of periplasmic proteins) proteases, also known as HtrA (high temperature requirement A) proteases, which are absent in yeast (Tanz et al., 2014), or oligopeptidases that degrade short polypeptides into amino acids in an ATP-independent manner (Käser and Langer, 2000).

The removal of the N-terminal fragment of a protein before it reaches its final destination due to the activity of specific processing peptidases is characteristic of a majority of mitochondrial proteins, and the best-studied enzyme possessing such an activity is mitochondrial processing peptidase (MPP) (Teixeira and Glaser, 2013). The cleavage of mitochondrial precursors by MPP may be followed by an additional trimming. In the mitochondrial matrix of yeast, the MPP-generated protein intermediates are sequentially cleaved by the mitochondrial intermediate peptidase (MIP) (Kalousek et al., 1988; Isaya et al., 1991) or the intermediate cleaving peptidase (Icp55; molecular weight 55 kDa) (Vögtle et al., 2009), leading to the formation of mature proteins. In yeast, MIP removes octapeptide residues located after the MPP cleavage site, and therefore, MIP is also designated as octapeptidyl aminopeptidase 1 (Oct1) (Gakh et al., 2002). Yeast Icp55 removes a single amino acid residue from the MPP-generated intermediates. In accordance with the N-end rule, the protein intermediate that acts as a substrate for these two proteases carries destabilizing amino acids at the N-terminus, and the second processing step converts this unstable precursor into a stable mature protein (Mossmann et al., 2012). Recently, homologs of yeast Oct1 (AtOCT1) and Icp55 (AtICP55) were identified in plant mitochondria (Carrie et al., 2015; Huang et al., 2015). While two types of substrates with the characteristic cleavage motifs were found for AtICP55, one similar to that observed in yeast and the second plant-specific, the AtOCT1 substrates from Arabidopsis displayed no consensus cleavage motif and lacked the classical -10R motif, which is characteristic of other eukaryotes (Carrie et al., 2015; Huang et al., 2015). The second processing step in yeast can also be carried out by an inner membrane peptidase (IMP) complex, consisting of two catalytic subunits Imp1 and Imp2, and the non-catalytic subunit Som1, as well as a rhomboid protease Pcp1 (processing of cytochrome c peroxidase). IMP cleaves off the hydrophobic sorting signals from proteins that have been previously processed by MPP and have been sorted to the intermembrane space (IMS). The yeast rhomboid Pcp1 carries out a second cleavage within the transmembrane region of its substrates, thus, releasing the mature proteins into the IMS. In plant mitochondria, only one ortholog of Pcp1 (rhomboid-like protein-AtRBL12) has been experimentally identified, but its substrates and mechanism of action remain unknown (Kmiec-Wisniewska et al., 2008). Moreover, an unusual two-step processing, in which both steps are carried out by MPP, has been reported in yeast (Branda et al., 1999) and plants (Kmiec et al., 2012).

In yeast mitochondria, it has been shown that besides the classical presequence peptidases (MPP, IMP, and Oct1), several other proteases show precursor processing activity. One of them is Atp23, a metallopeptidase located in the IMS, which mediates cleavage of the presequence of the mitochondrially encoded Atp6 after its insertion into the inner membrane (Osman et al., 2007; Zeng et al., 2007). Independent of its processing activity, Atp23 also exerts a chaperone function by promoting the assembly of Atp6 into the F0 complex of the F1F0 ATPase (Osman et al., 2007). Furthermore, it has been documented that Atp23 is involved in the turnover of the IMS protein Ups1, which regulates a proper distribution of phospholipids in the inner membrane (Potting et al., 2010).

Oma1 (overlapping activity with the *m*-AAA protease) was first discovered in yeast as an ATP-independent zinc metalloprotease, which was able to degrade misfolded membrane proteins in the absence of the *m*-AAA protease (Käser et al., 2003). In mammals, OMA1 is implicated in the processing of OPA1, a dynamin-like GTPase required for fusion of the mitochondrial inner membrane (Baker et al., 2014). Recently, it was shown that yeast Oma1 displays strongly enhanced activity in response to various stresses (Anand et al., 2013; Bohovych et al., 2014; Rainbolt et al., 2015; Richter et al., 2015). Furthermore, studies in yeast and metazoans have indicated that Oma1 is required for stabilization of the respiratory chain supercomplexes (RSCs) (Bohovych et al., 2015). Additionally, Oma1 was also shown to be involved in the regulation of TOR (target of rapamycin) signaling in yeast (Bohovych et al., 2016).

For many years, information on the ATP-independent proteolytic system in plant mitochondria was limited to the characterization of MPP. Recent studies on oligopeptidases (Kmiec et al., 2014), ICP55, and OCT1 proteases (Carrie et al., 2015; Huang et al., 2015) have started filling this knowledge gap. The goal of our study was to identify the similarities between the ATP-independent proteolytic systems in plants and yeast, and to ascertain the plant specific features of this system. Here, we have identified new Arabidopsis homologs of the yeast mitochondrial ATP-independent proteases: Imp1, Imp2, Atp23, and Oma1. We have analyzed them along with the earlier known plant ATP-independent mitochondrial proteases, AtICP55 and AtOCT1, in respect to their ability to functional complementation in yeast and their effect on Arabidopsis growth and development. We have demonstrated that out of all the proteases examined, only AtICP55 and AtOMA1 are able to rescue a yeast mutant phenotype lacking the respective protease, and that the lack of a single ATP-independent protease does not affect Arabidopsis growth and development, with the exception of AtOMA1. In order to gain further insights into the possible mitochondrial functions of the investigated proteases, functionality of OXPHOS in the respective null mutants was examined. The results suggested a significant role of AtOMA1 in the OXPHOS system, particularly in complex V, in contrast to the other Arabidopsis ATP-independent proteases investigated in this study.

## Materials and Methods

### Plant Material

*Arabidopsis thaliana* plants (Columbia ecotype, Col-0) were either grown in soil at 22°C with 70% humidity and 150 μmol⋅m^-2^⋅s^-1^ light with a 16-h light/8-h dark photoperiod (long day, LD) or in growth chambers under LD photoperiod at 22°C or 30°C (water cultures or agar plates). For growth in water cultures, ca. 200 sterilized seeds were added to 80 ml of growth medium (1/2 Murashige and Skoog medium (MS), 3% sucrose, 2 mM MES, 1.5 g/L Gamborg B5 vitamins (1000×), pH 5.7) and incubated on an orbital shaker at 80–100 rpm for 2 weeks. For growth on agar plates, sterile seeds were plated on 1/2 MS with 1.5% sucrose and 1% bactoagar, supplemented with either 300 mM mannitol or 0.5 μM paraquat. The T-DNA insertion mutant seeds (SALK_093517C, SALK_094274, SALK_077448, SALK_012114C, SALK_088054C, SAIL_135_G10, WiscDsLox507A03, GABI_893A04, SALK_080280, SALK_080262, SALK_080264, SALK_080272) were obtained from the Nottingham Arabidopsis Stock Centre (NASC). Homozygous mutants were selected by PCR on genomic DNA template using primers described in Supplementary Table S2. The position of the T-DNA insertion of the *A. thaliana* homozygous knockout plants was verified by sequencing. A lack of gene expression in the selected homozygous plants was determined by RT-PCR using primers listed in Supplementary Table S2. Amplification of the transcript for *ACT2* gene (At3g18780) was used as a control. Growth progression was analyzed according to Boyes et al. (2001).

### Plasmid Construction for Expression in Yeast, Protoplasts and Plants

For cloning of plant or yeast proteases, the full-length coding sequences were amplified by the Phusion Polymerase (Thermo Scientific) with primers listed in Supplementary Table S2 using as a template cDNA synthesized from the total RNA of wild-type plants or genomic DNA from the wild-type yeast cells, respectively. The PCR products were cloned into the Gateway vector pENTR-D-TOPO (Thermo Scientific) to generate the entry clones. For the transient expression in protoplasts, the entry clones were recombined into p2GWF7 (Joubès et al., 2004) to yield C-terminal GFP-tagged peptidases. For the stable expression of C-terminal GFP-tagged peptidases, the entry clones were recombined into pGWB551 (Nakagawa et al., 2007). For the stable expression of FLAG-tagged AtOMA1, the entry clone was recombined into pGWB511 (Nakagawa et al., 2007). For the stable expression of FLAG-tagged AtATP23, a sequence coding for a triple FLAG tag was added to the 5′ end of the AtATP23 coding sequence by PCR. The fusion PCR product was cloned into the Gateway vector pENTR-D-TOPO (Thermo Scientific) and the obtained clone was recombined into pGWB514 (Nakagawa et al., 2007). For the yeast complementation analysis, the entry clones were recombined into pVV209 (Van Mullem et al., 2003) to yield C-terminal HA-tagged peptidases. All constructs were verified by DNA sequencing and are listed in Supplementary Table S4.

### Protoplast Isolation, Transfection and Confocal Imaging

The protoplast isolation and transfection was performed according to Yoo et al. (2007). The photographs of protoplasts were taken with scanning confocal microscope (Olympus and Zeiss) equipped with a 40X objective NA = 0.95, using the excitation wavelength 488 nm for GFP and 561 nm for MitoTracker CMXRos (Life Technologies). Images of protoplasts were processed and analyzed with ImageJ software^1^.

### Plant Transformation

The transgenic Arabidopsis *atp23flag^OE^, oct1flag^OE^, oma1flag^OE^* and *imp1agfp^OE^* plants were generated via vacuum infiltration (Clough and Bent, 1998) using *Agrobacterium tumefaciens* strain LBA4404. The selection of transformants was performed on 1/2 MS medium supplemented with 1.5% sucrose and 30 μg/ml hygromycin.

### Yeast Strains, Transformation and Complementation Assay

Yeast strains *imp1Δ* (*IMP1::kanMX4*), *imp2Δ* (*IMP2::kanMX4*), *oct1Δ* (oct1::*kanMX4*), *icp55Δ* (*ICP55::kanMX4*), and *oma1Δ* (OMA1::*kanMX4)* were isogenic to BY4742 and obtained from Euroscarf ^2^. The CW3 strain (*atp23*Δ::*HIS3MX6*), isogenic to W303-1B, was kindly provided by prof. Thomas Langer (University of Cologne). The strain *coa2Δ* was generated by PCR-based gene replacement method using *natMX4* cassette amplified from plasmid pAG25 (Goldstein and McCusker, 1999). The disruption of the *COA2* gene in *oma1Δ* was generated using the *HIS3MX6* cassette amplified from plasmid pFA6a-His3MX6 (Longtine et al., 1998). PCR primers used to amplify the cassette and verify the disruption are listed in Supplementary Table S3. The strains were transformed using the lithium acetate method according to Gietz et al. (1995). For the functional complementation assay yeast cells were grown overnight in a liquid complete synthetic drop-out medium (0.7% yeast nitrogen base without amino acids, 2% glucose) lacking appropriate auxotrophic markers. Serial dilutions of the same number of cells were plated on YPD (1% yeast extract, 2% peptone, 2% glucose) or YPG (1% yeast extract, 2% peptone, 2% glycerol) plates and incubated at 30°C or 37°C for 3 days.

### Mitochondria and Chloroplast Isolation

For SDS-PAGE and BN-PAGE experiments, mitochondria were isolated from Arabidopsis water cultures according to Day et al. (1985). Typically, 1 mg of mitochondrial protein was obtained from each preparation. Isolation of yeast mitochondria was performed according to Daum et al. (1982). Isolation of chloroplasts was performed using Chloroplast Isolation Kit (Sigma Aldrich). Protein concentration was determined using the DC Protein Assay (Bio-Rad).

### Fractionation of Mitochondria

Hundred microgram of mitochondrial proteins from the *oct1flag^OE^*, *imp1agfp^OE^*, *atp23flag^OE^* lines were suspended in 200 μl of 0.1 M Na_2_CO_3_, pH 11, and the suspension was sonicated three times for 10 s on ice, followed by centrifugation at 100000 ×*g* for 60 min. The resulting pellet (membrane fraction) and supernatant (soluble fraction) were directly dissolved in the electrophoresis sample buffer. Mitochondria from *oma1flag^OE^* were suspended in 200 μl of 0.1 M Na_2_CO_3_, pH 10.7, and directly centrifuged. Afterward, the obtained pellet and supernatant were dissolved in the electrophoresis sample buffer with the addition of 8 M urea.

### BN-PAGE and In-gel Activity Assays

One-dimensional blue-native gel electrophoresis (BN-PAGE) and catalytic staining of mitochondrial respiratory chain complexes were performed as described in Kolodziejczak et al. (2007). Bands were quantified using ImageJ software^1^. Two-dimensional BN-PAGE was carried out as described in Piechota et al. (2010).

### SDS-PAGE and Immunoblot Analysis

Total protein extracts (30 μg) or mitochondrial proteins (30 μg) were resolved on SDS-PAGE according to Laemmli (1970) using 12% gel (unless otherwise indicated) and then transferred onto nitrocellulose or PVDF (Bio-Rad) membrane. Blots were probed using the primary antibodies listed in Supplementary Table S5. Anti-rabbit or anti-mouse secondary antibodies conjugated to horseradish peroxidase were used, and the signal was visualized with the enhanced chemiluminescent ECL reagent (Advansta) and the GBox imager (Syngene).

### Quantitative Real-time PCR Analysis

Total RNA was extracted from leaves of 4-week-old plants using GeneMATRIX Universal RNA Purification Kit (EURx). Reverse transcription analysis and the quantification of mRNA were performed by quantitative RT-PCR as previously described (Kwasniak et al., 2013). The wild-type plants served as a calibrator, and the *ACT2* gene (At3g18780) was used as a reference. The data were analyzed using the LightCycler software version 4.0 (Roche Diagnostics). All primers used for qRT-PCR are listed in Supplementary Table S6.

### Respiration Assays

Respiration of isolated mitochondria of 2-week-old wild-type and *oma1-1* seedlings grown under LD, 22°C, was recorded using a Clark-type oxygen electrode (Hansatech). The measurements were performed at 25°C in an incubation buffer containing 0.3 M mannitol, 10 mM TES-KOH (pH 7.5), 3 mM MgSO_4_, 10 mM NaCl, 5 mM KH_2_PO_4_ and 0.1% BSA. Succinate (SA) at 5 mM and NADH at 1 mM were the respiratory substrates. 0.5 mM ATP and 2 mM SHAM were added to the incubation medium to ensure full activity of SDH and to block AOX-mediated oxygen consumption, respectively. The ADP-stimulated respiration (state 3) was measured in the presence of 0.2 mM ADP. To estimate the production of ATP by isolated mitochondria, oligomycin A (2 μg/ml) was added to inhibit ATP synthesis.

### Statistical Analysis

Statistical analyses were conducted using Microsoft Excel. Error bars represent the standard deviation (SD) of at least three independent experiments. Statistical significance was assessed using Student’s *t*-test and considered significant at *p* ≤ 0.05.

## Results

### Mitochondrial Localization of Arabidopsis Homologs of Yeast ATP-Independent Peptidases

In a previous report, through sequence comparisons, we have shown that the Arabidopsis genome contains homologs of all known yeast ATP-independent peptidases, with the exception of yeast thiol-aminopeptidase (Lap3) (Kwasniak et al., 2012). Besides the presence of already known plant ATP-independent peptidases, such as MPP, RBL12, and PREP, an *in silico* analysis revealed the presence of a homolog each of yeast Oct1 (At5g51540), Icp55 (At1g09300), Atp23 (At3g03420), Oma1 (At5g51740), and Prd1 (At5g65620), and six homologs of Imp (At1g53530, At3g08980, At1g23465, At1g29960, At1g06870, and At2g31140) in the *A. thaliana* genome. Recently, the predicted homologs of yeast Prd1 (AtOOP), Icp55 (AtICP55), and Oct1 (AtOCT1) were identified experimentally in Arabidopsis mitochondria (Kmiec et al., 2013; Carrie et al., 2015; Huang et al., 2015). In the present study, we investigated the mitochondrial localization of the remaining expected ATP-independent proteases, namely Atp23 (AtATP23), Oma1 (AtOMA1), and selected Imp homologs (AtIMP1a, AtIMP1b, and AtIMP2). Additionally, in order to explore the possible targeting of AtOCT1 into chloroplasts (Kleffmann et al., 2004), we decided to determine the subcellular targeting of AtOCT1, even though its mitochondrial localization was recently proven by *in vitro* import into mitochondria isolated from Arabidopsis (Carrie et al., 2015). We excluded three Imp homologs (At1g29960, At1g06870, and At3g08980) from this study, considering their non-mitochondrial localization based on prediction studies and proteomic data (Feiz et al., 2006; Ni et al., 2010; Hooper et al., 2014). Taking into account the presence of the diagnostic motif RX5P in Imp1 and NX5S in Imp2 (Burri et al., 2005), we were able to distinguish the plant homologs of yeast Imp1 (AtIMP1a and AtIMP1b) and Imp2 (AtIMP2) (Supplementary Figure S1). The nomenclature of the plant proteases analyzed in this study with reference to yeast enzymes is given in **Table 1**.

**Table 1:** Nomenclature, localization and functional complementation in yeast Arabidopsis ATP-independent proteases examined in this work.

	Yeast	Plants			
ATP-independent proteases	Name	Locus	Name	Localization	Functional complementation in yeast
	**Imp1**	At1g53530	**AtIMP1a**	**Mitochondrion (this study)**	Negative
		At1g23465	**AtIMP1b**	**n.d.**	n.d.
	**Imp2**	At2g31140	**AtIMP2**	Mitochondrion^∗^ (Klodmann et al., 2011) vacuole^∗^ (Jaquinod et al., 2007) **Mitochondrion (this study)**	Negative
	**Atp23**	At3g03420	**AtATP23**	Mitochondrion^∗^ (Klodmann et al., 2011) **Mitochondrion (this study)**	Negative
	**Oma1**	At5g51740	**AtOMA1**	Mitochondrion^∗^ (Klodmann et al., 2011) **Mitochondrion, (this study)**	**Positive**
	**Oct1**	At5g51540	**AtOCT1**	Mitochondrion (Carrie et al., 2015) **Mitochondrion, (this study)**	Negative
	**Icp55**	At1g09300.1	**AtICP55.1**	Mitochondrion (Carrie et al., 2015; Huang et al., 2015)	**Positive**

*^∗^Proteomics data; n.d- parameters not determined since we were not able to confirm mitochondrial localization of AtIMP1b.*

**FIGURE 1:**
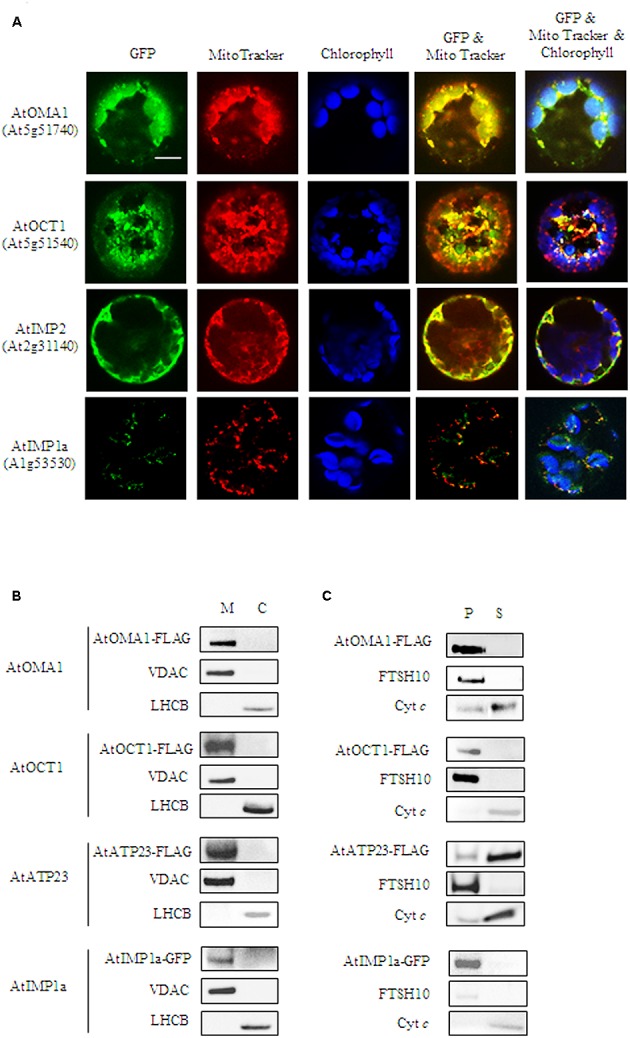
Mitochondrial localization of AtOMA1, AtOCT1, AtIMP1a, AtIMP2, and AtATP23 proteases. **(A)** Fluorescence imaging of Arabidopsis protoplast expressing the full-length sequence of the protease fused to GFP. The localization of AtOMA1, AtOCT1 and AtIMP2 has been performed using transient expression, while AtIMP1a constitutive expression of the GFP-fusion protein. Mitochondria were detected by staining with Mito Tracker while chloroplast were distinguished by the distribution of their chlorophyll autofluorescence. The fluorescent signals of GFP was merged with Mito Tracker or both Mito Tracker and chlorophyll autofluorescence. Scale bar = 10 μm. **(B)** Detection of FLAG-tagged AtOMA1, AtOCT1, AtATP23 and GFP-tagged AtIMP1a in mitochondria (M), and chloroplasts (C) isolated from the stable *A. thaliana* transformants. **(C)** Fractionation of mitochondria isolated from the stable *A. thaliana* transformants as in **(B)**. Abbreviations: (S) soluble protein fraction, (P) pellet - membrane protein fraction of mitochondria. Antibodies used in **(B,C)** were anti-FLAG, anti-GFP, anti-VDAC1-5 (mitochondrial marker), anti-LHCB (chloroplast marker), anti-FTSH10 (marker of mitochondrial inner membrane), anti-Cyt *c* (Cytochrome *c*, marker of mitochondrial intermembrane space).

To verify the predicted mitochondrial localization, two approaches were used. In the first approach, constructs containing a green fluorescent protein (GFP) fused to the C-terminus of the full-length coding sequences of the proteases were used for transient expression in Arabidopsis protoplasts. Colocalization of the GFP signal with a mitochondria-specific fluorescent probe MitoTracker CMXRos was observed for AtOMA1-GFP, AtOCT1-GFP, and AtIMP2-GFP, suggesting the mitochondrial localization of these fusion proteins (**Figure 1A**). However, using transient expression, we were unable to detect the GFP signal from AtIMP1a-GFP and AtIMP1b-GFP fusion proteins, whereas the AtATP23-GFP fusion protein tended to aggregate into clumps. In the second approach, we decided to generate Arabidopsis transgenic lines constitutively expressing C-terminal FLAG-tagged full-length variants of AtOMA1, AtOCT1, and AtATP23 proteases and C-terminal GFP-tagged full-length variants of AtIMP1a, AtIMP1b, and AtIMP2. We were successful in generating all the lines, with the exception of lines expressing AtIMP1b-GFP and AtIMP2-GFP. The expression of fusion proteins was monitored by immunodetection (**Figure 1B**). In the case of AtIMP1a-GFP fusion protein, both fluorescence microscopy (**Figure 1A**) and immunoblotting (**Figure 1B**) were used. Detection of the tags exclusively in mitochondria, but not in chloroplasts, fully confirmed the results of the transient expression assay for AtOMA1 and AtOCT1, and clearly revealed the mitochondrial localization of the tagged variants of AtATP23 and AtIMP1a (**Figure 1B**). **Table 1** summarizes the results obtained from the localization experiment.

Next, we fractionated mitochondria isolated from Arabidopsis lines constitutively expressing the tagged fusion proteases in order to examine if the enzymes being investigated were soluble or membrane localized. Similar to their yeast counterparts, AtATP23 behaved like a loosely associated with the inner membrane Cyt *c*, whereas AtOMA1 and AtIMP1a were detected in the membrane protein fraction (**Figure 1C**). Surprisingly, AtOCT1 was also found along with the membrane proteins. This result is in contradiction with the matrix localization of the AtOCT1 homolog in yeast mitochondria (**Figure 1C**) (Isaya et al., 1994).

### Functional Complementation in Yeast Mutants

**FIGURE 2:**
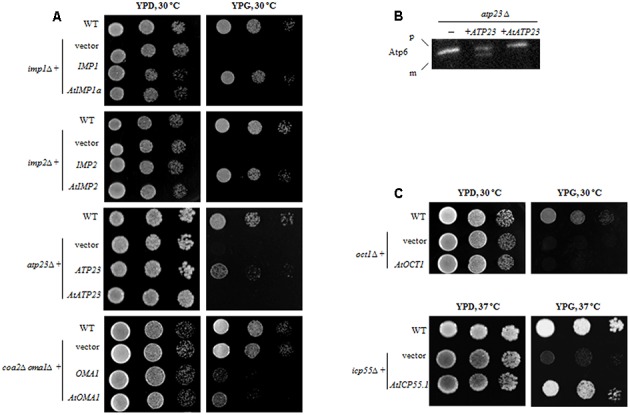
Functional complementation of yeast mutants lacking the respective ATP-independent protease by plant homolog. **(A)** Yeast deletion mutants were transformed with plasmid carrying a full-length coding sequence of a plant protease or a coding sequence of yeast protease as a positive control. Ten-fold serial dilutions of yeast cells were spotted onto the plates with rich medium containing 2% glycerol (YPG) and incubated at indicated temperature for 3 days. Wild-type and yeast deletion mutants carrying empty plasmid were spotted as a control (vector). **(B)** *atp23Δ* yeast mutants were transformed with plasmid carrying either plant *AtATP23* or yeast *ATP23* gene (positive control). Mitochondria from the yeast cells were probed with anti-Atp6 antibodies. p, precursor form of Atp6; m, mature form of Atp6; “–” untransformed *atp23Δ* cells. **(C)** Yeast deletion mutants were transformed with plasmid carrying a full-length coding sequence of a plant protease. Plate assay as in **(A)**.

To check whether the function of Arabidopsis mitochondrial ATP-independent proteases is similar to their yeast counterparts, a functional complementation assay was conducted in yeast. We tested the Arabidopsis proteases identified in this study (AtIMP1a, AtIMP2, AtATP23, and AtOMA1) (**Figures 2A,B**) along with AtOCT1 and AtICP55, the ATP-independent proteases described earlier by Carrie et al. (2015) and Huang et al. (2015) (**Figure 2C**). The phenotypes of yeast mutants lacking the respective protease growing on non-fermentable carbon sources were described in the following publications: *imp1Δ* and *imp2Δ* (Burri et al., 2005), *oct1Δ* (Isaya et al., 1994), *atp23Δ* (Osman et al., 2007; Zeng et al., 2007), *oma1Δcoa2Δ* (Khalimonchuk et al., 2012), and *icp55Δ* (Vögtle et al., 2009). After transformation, the presence of HA-tagged Arabidopsis proteases in yeast mutant mitochondria was confirmed by immunoblotting (Supplementary Figure S2). However, the Arabidopsis ATP23 protein could not be stably expressed until it was fused with a mitochondrial targeting sequence derived from the yeast cytochrome b2 protein (Cyb2) (Supplementary Figure S2B).

When yeast knockout mutants lacking the *IMP1* or *IMP2* gene were transformed with constructs carrying their plant counterparts *(AtIMP1a* or *AtIMP2)*, the defect in respiratory growth could not be restored in yeast cells grown in a medium containing glycerol as the carbon source (YPG medium) (**Figure 2A**). Similarly, in the functional complementation experiments, we found that the AtATP23 protein did not restore respiratory function of the *atp23Δ* yeast mutant (**Figure 2A**) and was also unable to process yeast Atp6 (**Figure 2B**). Furthermore, the plant homolog of Oct1 could not restore respiratory function in the *oct1Δ* yeast mutant (**Figure 2C**), suggesting a functional divergence between the plant and yeast proteins, which has been postulated previously as well (Carrie et al., 2015).

In contrast to the above results, AtOMA1 and AtICP55 were able to substitute for their yeast homologs in the functional complementation assays. It has been reported that a depletion of Oma1 results in progressive impairment of the respiratory function in aging *oma1Δ* mutants (Bohovych et al., 2015). However, the same results were not obtained in repeat experiments. Therefore, we decided to use another functional complementation system, the yeast mutant lacking both Oma1 and Coa2 (the assembly factor for Cox1 biogenesis required during cytochrome *c* oxidase maturation). It has been demonstrated that Oma1 protease mediates degradation of newly synthesized, unassembled Cox1 in cells lacking the Coa2 assembly factor (Khalimonchuk et al., 2012). Thus, the lack of Oma1 in *coa2*Δ leads to stabilization of the unassembled Cox1 and restores respiratory function. In other words, while the *coa2*Δ mutant is not able to grow on glycerol, the double mutant *coa2*Δ*oma1*Δ displays growth on media containing non-fermentable carbon sources. As can be inferred from **Figure 2A**, the plant homolog of Oma1 in *coa2*Δ*oma1*Δ was able to partially replace the degradation function of the yeast protease. It has also been shown that the AtICP55 protease performs a function similar to its yeast counterpart, which is critical for the stability of mitochondrial proteins (Carrie et al., 2015; Huang et al., 2015). Our complementation studies are in agreement with this previous finding since AtICP55 was able to restore respiration in impaired yeast cells lacking ICP55 (**Figure 2C**).

### Selection of T-DNA Insertion Lines

To investigate the impact of mitochondrial ATP-independent proteases on plant growth and development, we selected homozygous lines with a T-DNA insertion in the gene of interest. PCR analysis was performed in order to select two homozygous lines each for AtATP23, AtOMA1, and AtICP55, and one line each for AtIMP1a and AtOCT1 (Supplementary Figure S3A). Localization of T-DNA insertion sites in the respective genes is shown schematically in Supplementary Figure S4. We observed that the insertion lines for AtICP55 and AtOCT1 identified by us were different from the ones selected by Carrie et al. (2015) and Huang et al. (2015). We could not identify T-DNA insertions in any of the AtIMP2 lines examined (SALK_080262, SALK_080280, SALK_080264, and SALK_080272) (Supplementary Figure S3B). RNA analysis revealed that the full-length transcript was not synthesized in four out of the eight selected homozygous lines, including *imp1a-1* (SALK_094274), *oma1-1* (SALK_088054C), *oct1-1* (SALK_077448), and *icp55-1* (GABI_893A04) (Supplementary Figure S3C). All these null mutants carried a T-DNA insertion in the coding region (Supplementary Figure S4) and were used for further analysis.

### Characterization of T-DNA Insertion Lines

**FIGURE 3:**
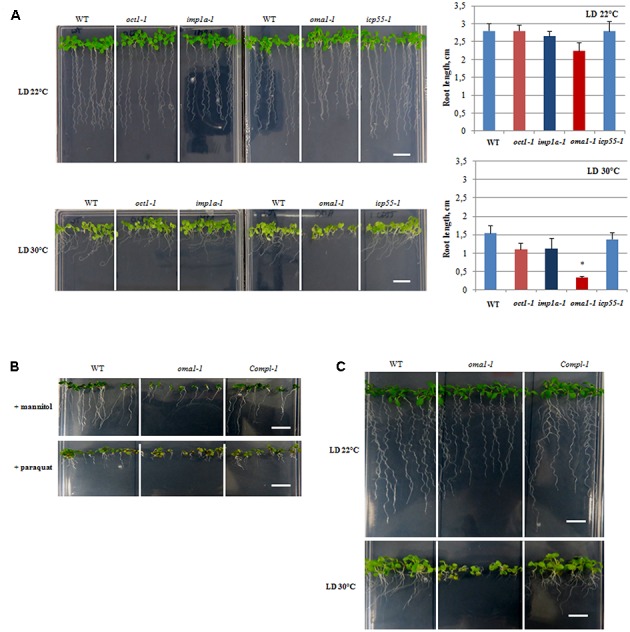
Morphology of 10-day-old seedlings. **(A)** WT, *oct1-1*, *imp1a-1*, *oma1-1*, *icp55-1* plants grown under long day (LD) 22°C and 30°C. The length of the roots of 10-day-old seedlings. Mean values ± SD from at least three individual plants. ^∗^*p* ≤ 0.05. **(B)** WT, *oma1-1* and the complement line (*Compl-1*) grown in the presence of 300 mM mannitol or 0.05 μM paraquat. **(C)** WT, *oma1-1* and the complement line (*Compl-1*) grown under (LD) 22°C and 30°C; bar = 1 cm.

A growth stage-based progression analysis was performed for *imp1a-1, oma1-1*, *oct1-1*, and *icp55-1* growing under optimal conditions, following the method of Boyes et al. (2001). No significant developmental alterations were observed in any of the examined knockout lines (Supplementary Figures S5A,B). Analysis of seedling morphology revealed that only *oma1-1* displays a slight reduction in root length (**Figure 3A**, Supplementary Figure S5C). The Arabidopsis eFP Browser indicates that the expression of *AtOMA1* is induced by heat stress (Winter et al., 2007). Therefore, we examined the growth of *oma1*-*1* plants as well as other null mutants under moderate heat stress (LD, 30°C) (**Figure 3A**). Growth of *imp1a-1, oct1-1*, and *icp55-1* was indistinguishable from wild-type plants. However, *oma1-1* revealed strong phenotypic differences under this condition: an overall decrease in seedling size with an 80% reduction in root length compared with the wild-type plants (**Figure 3A**). We also found that plants lacking AtOMA1 exhibited increased sensitivity to osmotic stress (imposed by high concentrations of mannitol) and oxidative stress (induced by paraquat) (**Figure 3B**). To confirm that the observed morphological alterations in *oma1-1* were caused due to the lack of AtOMA1 protein, we performed a complementation assay by transforming the *oma1-1* line with the full-length cDNA of At5g51740 (Supplementary Figure S3D). Analysis of seedling morphology under optimal as well as stress conditions indicated recovery of the wild-type phenotype in complemented lines (**Figure 3C**), thus, confirming that the phenotype of *oma1-1* mutant is caused solely by the lack of AtOMA1.

### The Oxidative Phosphorylation (OXPHOS) System in Arabidopsis Mitochondria Lacking Functional AtIMP1a, AtOMA1, AtOCT1, and AtICP55 Proteases

We investigated the impact of the examined proteases on the functionality of the OXPHOS system by using a combination of blue-native polyacrylamide gel electrophoresis (BN-PAGE) and in-gel histochemical staining to detect the enzymatic activities of complexes I, IV, and V. This analysis revealed that the loss of AtIMP1a, AtOCT1, or AtICP55 did not cause significant changes in activity and protein level of the investigated complexes in plants grown under optimal conditions (Supplementary Figure S6).

**FIGURE 4:**
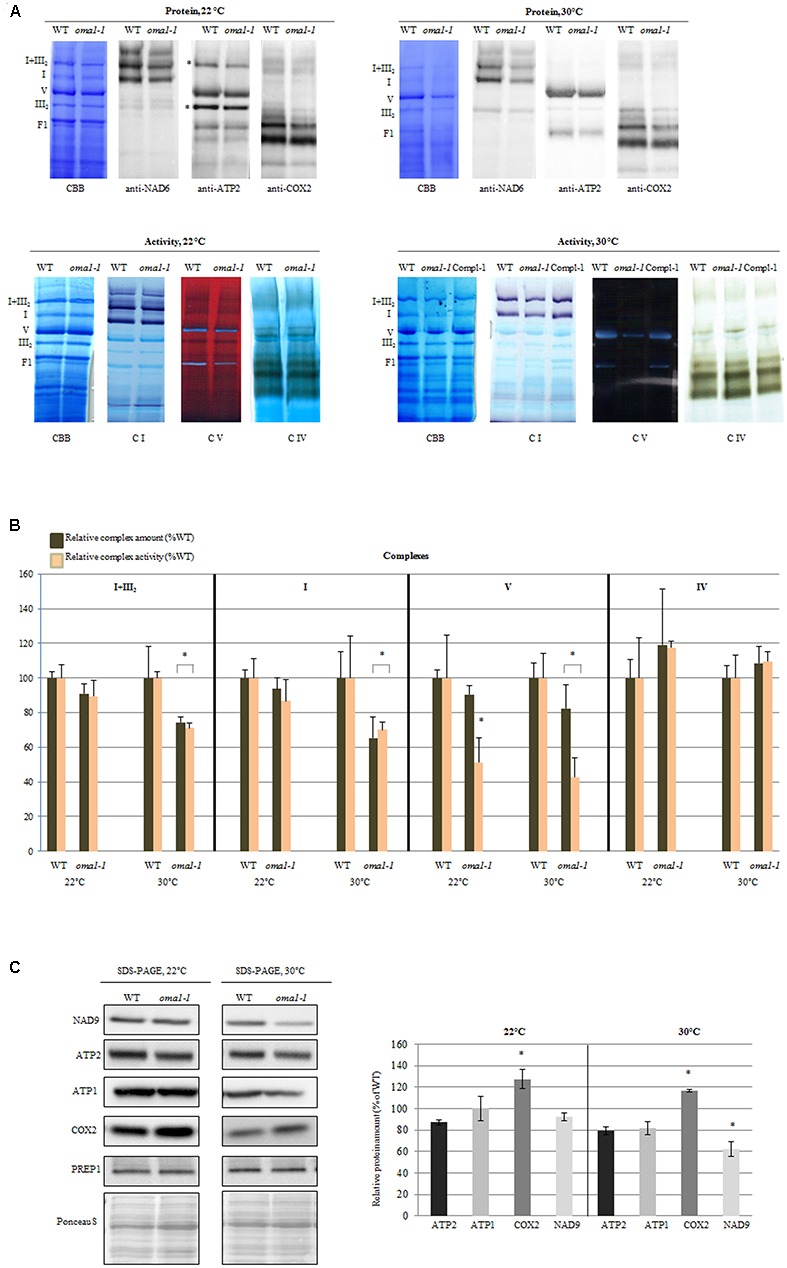
Activity and abundance of the OXPHOS complexes and their subunits in *oma1-1* mitochondria. **(A)** Immunodetection, Coomassie (CBB) staining and in-gel enzyme activity of mitochondrial respiratory chain complexes separated by blue-native polyacrylamide gel electrophoresis (BN-PAGE). Mitochondria were isolated from 14-day-old WT and *oma1-1* plants grown under optimal conditions (LD, 22°C) or at elevated temperature (LD, 30°C). **(B)** Quantification of the activity and abundance of the analyzed complexes. The protein level of complexes I and V was quantified based on CBB staining and immunodetection with antibodies directed against selected subunits. The abundance of complex IV was quantified solely based on signal detection from antibody directed against COX2. In each experiment, values of relative activity and protein abundance were calculated as percentage of volume determined for wild-type plants (set to 100%). **(C)** Western blot analysis of the subunits of complex I, IV and V in isolated mitochondrial fraction using SDS-PAGE. Representative immunoblots are shown. Protein amount was quantified densitometrically and values are given as percentage of the value obtained for wild-type plants (set as 100%). Mean values ± SD from at least three independent experiments are shown. The two-tailed Student’s *t*-test was used to determine if the differences between wild-type and *oma1-1* mutants are statistically significant.^∗^*p* ≤ 0.05.

The only exception was *oma1-1* mutant, which showed an evident reduction (∼50%) in the activity of complex V (even though the total protein level remained unchanged) as compared with the wild-type plants (**Figures 4A,B**). The functionality of complex V was also affected when the *oma1-1* plants were grown at 30°C (**Figure 4**), resulting in a decrease in activity by 60% and a slight but statistically relevant decline in the total amount of protein (**Figures 4A,B**). Interestingly, protein level and activity of complex IV, both were slightly higher in the mutant (**Figures 4A,B**), particularly under optimal conditions. In contrast, reduction in the activity of complex I and supercomplex I + III_2_, which was correlated with decreased protein level, was observed exclusively under moderate heat stress conditions in *oma1-1*. Furthermore, analysis using BN/SDS-PAGE followed by immunoblotting with antibodies directed against subunits of the examined complexes led to similar results on the abundance of supercomplex I + III_2_ and complexes I, IV, and V in *oma1-1* mitochondria under stress conditions (Supplementary Figure S7). It should be emphasized that protein amount and activity of the OXPHOS complexes were restored to the wild-type levels in the complemented line of *oma1-1*, thus, confirming that the observed defects resulted from AtOMA1 deficiency (**Figure 4A**).

There could have been many probable reasons for the alterations in the protein amount of complexes. To better understand this issue, we quantified the abundance of mRNAs encoding representative components of complexes I, IV, and V. The levels of these transcripts were generally unaffected by the loss of AtOMA1 under both optimal and moderate heat stress conditions, except for a slight increase in *ATP2-3* under optimal conditions, *coxII* under elevated temperature, and *CA2* under both conditions (Supplementary Figure S8). These findings clearly imply that the reduction in the abundance of OXPHOS complexes in the *oma1-1* mutant was not due to changes in the transcript levels. Similarly, accumulation of complex IV in *oma1-1* was not due to transcriptional induction of genes encoding its subunits, since a higher level of only one transcript (*coxII*) was observed, and that too, exclusively under elevated temperature conditions.

In contrast to BN-PAGE or BN/SDS-PAGE, detection of proteins using SDS-PAGE not only allows the visualization of complex-assembled subunits, but also of unassembled subunits. The western blot experiment presented in **Figure 4C** indicates that in mitochondria lacking AtOMA1, the abundance of NAD9 (complex I) as well as ATP1 and ATP2 (complex V) was unaltered under optimal growth conditions, whereas a decline in the level of these subunits was found under stress conditions. On the other hand, the protein amount of complex IV subunit consistently increased under both conditions. Thus, the SDS-PAGE (complex subunit) and BN-PAGE (whole complex) results indicated similarities in the abundance of OXPHOS complexes.

**FIGURE 5:**
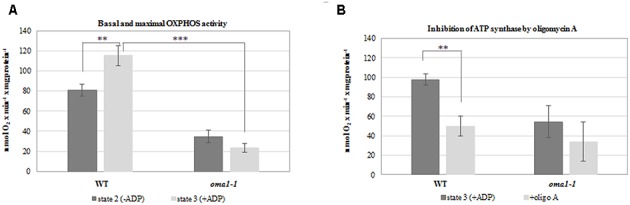
Oxygen consumption rates (nmol O_2_ × min^-1^ × mg of protein^-1^) by mitochondria isolated from 14-day-old wild-type and *oma1-1* plants grown under optimal conditions (LD, 22°C). **(A)** Basal respiration rate (state 2) and maximal (state 3) OXPHOS activity. **(B)** The ATP production rate after inhibition of ATP synthase with oligomycin A. Mean values ± SD from three independent experiments are shown. ^∗∗^*p* ≤ 0.005; ^∗∗∗^*p* ≤ 0.0005.

To confirm the aberrant functionality of OXPHOS in *oma1-1*, we measured the rate of oxygen uptake by mitochondria isolated from wild-type and *oma1-1* plants grown under optimal conditions, with succinate and NADH as the respiratory substrates. We observed a significantly lower basal respiration rate (state 2) in *oma1-1* in comparison with the wild-type mitochondria (**Figure 5A**). Furthermore, while the respiration rate increased in wild-type mitochondria on addition of ADP (state 3), the *oma1-1* mitochondria were unable to respond to this treatment (**Figure 5A**). In addition, the ATP production rate (measured as the respiration rate after inhibition of ATP synthase by oligomycin A) was lowered in the *oma1-1* mutant in comparison with the wild-type (**Figure 5B**). Thus, our data indicated that *oma1-1* mitochondria showed impaired ATP production and were not capable of reaching full respiratory capacity, even though the electron transfer chain was stimulated to work at a maximum rate.

### Relative Transcript and Protein Abundance of Mitochondrial Proteases in the Studied Mutants

**FIGURE 6:**
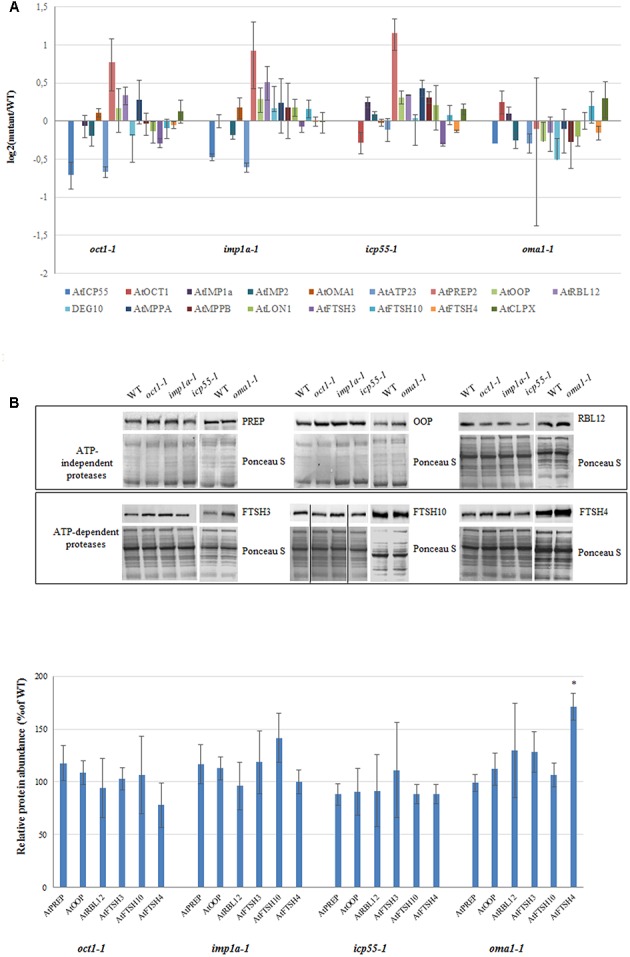
Expression level for selected genes encoding mitochondrial proteases in 4-week-old *oct1-1, imp1a-1, icp55-1* and *oma1-1* mutants grown under LD, 22°C, compared to wild-type plants. **(A)** The transcript level of mitochondrial ATP-independent and ATP-dependent proteases. Analysis was performed by quantitative real-time PCR. Relative abundance of transcripts is expressed as log2 ratios. Mean values ± SD from four independent experiments are shown. **(B)** Abundance of PREP (PREP1 and PREP2), OOP, RBL12, FTSH3, FTSH10 and FTSH4 proteases in mitochondria of WT and studied mutants. Ponceau S serves as a loading control. 30 μg of mitochondrial protein was loaded on gel. Protein amount was quantified densitometrically and values are given as percentage of the value obtained for wild-type plants (set as 100%). Mean values ± SD from three independent experiments are shown. ^∗^*p* ≤ 0.05.

It is possible that the loss of a single protease is compensated by the functions of the other proteases, and therefore, no phenotypic changes are visible. This compensation may result from redundancy between mitochondrial proteases and/or induction of other proteases at the transcript and/or protein level. To check if this second possibility explains the observed lack of altered phenotype in almost all of the investigated mutants under optimal growth conditions, the transcript abundance of a bulk of known mitochondrially localized proteases was quantified using real-time PCR (**Figure 6A**). Surprisingly, no clear transcriptional response was observed from genes encoding different mitochondrial proteases in the mutants lacking a single protease, with the exception of *PREP2*, the targeting peptide degrading protease/oligopeptidase (Kmiec et al., 2014), which was slightly up-regulated in three out of the four mutants analyzed (**Figure 6A**). Next, the protein level of several mitochondrial proteases was evaluated by immunoblotting using available antibodies (**Figure 6B**). No significant changes were observed in any mutant line, including PREP, except for FTSH4 in *oma1-1* and FTSH10 in *imp1a-1*, which showed an increase of approximately 50 and 30%, respectively (**Figure 6B**).

## Discussion

Intramitochondrial proteolysis is crucial for maintaining the functional state of the mitochondrial proteome. It was reported that a lack of some mitoproteases strongly influenced mitochondrial processes as well as overall plant growth and development (Rigas et al., 2009; Kmiec et al., 2013; Zhang et al., 2014; Smakowska et al., 2016). The main aim of this study was to identify such proteases functional in the ATP-independent proteolytic system, but first, we needed to verify their predicted mitochondrial localization.

In the present study, we have experimentally proven the presence of four homologs of yeast ATP-independent mitochondrial proteases in Arabidopsis mitochondria: AtIMP1a, AtIMP2, AtATP23, and AtOMA1 (**Table 1**). In yeast, the Imp protease consists of two catalytic subunits, whereas the Arabidopsis genome contains six orthologs for this gene. Taking into account the presence of the sequence motif characteristic of these proteins (Burri et al., 2005), we were able to distinguish the plant homologs of yeast Imp1 (AtIMP1a and AtIMP1b) and Imp2 (AtIMP2). Mitochondrial targeting could be confirmed only for AtIMP1a and AtIMP2. However, we do not exclude the possibility that AtIMP1b encodes mitochondrial proteins as well.

We also showed that like their yeast counterparts, AtIMP1a and AtOMA1 are integral membrane enzymes, whereas AtATP23 is a membrane-associated protein. Surprisingly, we found that the AtOCT1 protease is an integral membrane protein, in contrast with the matrix localization of yeast and human homologs. This last finding is supported by analysis using the OCTOPUS software (Viklund and Elofsson, 2008^3^), which predicts a hydrophobic region within the C-terminal end of AtOCT1 sequence. This region is not present in the yeast and mammalian counterparts (Supplementary Table S1).

The Arabidopsis proteases identified in this work as mitochondrial proteins (AtIMP1a, AtIMP2, AtATP23, and AtOMA1) as well as the two ATP-independent mitochondrial proteases (AtOCT1 and AtICP55) identified earlier (Carrie et al., 2015; Huang et al., 2015), were analyzed by heterologous expression in *Saccharomyces cerevisiae*. Out of the six plant homologs of the yeast ATP-independent proteases, only two, AtICP55 and AtOMA1, were able to substitute the absence of their yeast counterparts. Based on successful functional complementation in yeast, we assumed that AtICP55, like its yeast homolog, stabilizes mitochondrial proteins by the removal of single amino acids from MPP-processed proteins (Vögtle et al., 2009), whereas AtOMA1 could degrade misfolded membrane proteins (Khalimonchuk et al., 2012). Stabilization of the mitochondrial proteome by Arabidopsis ICP55 was recently documented by two independent studies monitoring the amino-terminal peptides (Carrie et al., 2015; Huang et al., 2015).

To evaluate the importance of ATP-independent proteases in overall plant development and morphology, and to test their role in the mitochondrial OXPHOS system, we decided to use a reverse genetic strategy. However, null T-DNA insertion mutants could be identified only for AtIMP1a, AtOMA1, AtOCT1, and AtICP55 (**Table 1**). Except for *oma1-1* mutant, the tested knockout plant lines did not show noticeable alterations in growth and development in comparison with the wild-type plants. The lack of AtOMA1 did not cause strong morphological changes under optimal growth conditions; however, roots were slightly shorter in length. In contrast, a drastic retardation in root growth was found under stress conditions. Similarly, out of all the null mutants examined, only the absence of AtOMA1 affected OXPHOS functionality. The lack of visible phenotypic alterations could be explained by functional redundancy and/or functional complementation among plant mitochondrial proteases. We did not find any evidence indicating a clear functional response at the transcript and protein level in mutants lacking phenotypic alterations, suggesting some degree of redundancy within the mitochondrial proteolytic machinery. Thus, in contrast to yeast, plant mitochondrial bioenergetic function and overall growth cannot be disrupted by the loss of a single peptidase such as AtIMP1a, AtOCT1, or AtICP55. On the other hand, our data indicates the necessity of AtOMA1 for proper functioning of the OXPHOS complexes, particularly the complex V, and for Arabidopsis root growth.

There are no previous reports indicating the influence of OMA1 protease on the activity of complex V, suggesting that this role of AtOMA1 is limited to plants. However, it was reported that in mammals, a removal of the OMA1 proteolytic substrates, C11orf83 and OPA1 (optic atrophy 1), causes perturbation in complex V assembly by destabilizing the F0 subunit (Patten et al., 2014; Desmurs et al., 2015). In contrast, our findings indicate that the deficiency in the activity of complex V in *oma1-1* under optimal growth conditions is not caused by a defect in the assembly/stability; rather, it is caused by its catalytic deficiency. This conclusion is based on the observation that a severe reduction in the activity of complex V was associated with a nearly normal abundance of this complex at the protein level (**Figure 4**). This distinctive disorder may be caused by accumulation of the complex V inhibitor, which binds to specific locations in the complex, inhibiting its action, due to the lack of proteolytic activity of AtOMA1.

Under moderate stress conditions, the activity of complex V was affected even further in *oma1-1* plants. This additional decline in activity was proportional to the decrease in the protein level of complex V, implying that under stress conditions, the deficiency in complex V functionality in *oma1-1* is caused by the inhibition of complex activity, and additionally, by defects in the complex assembly/stability (**Figure 4**). A correlation between amount of protein and activity levels of complex I and supercomplex I + III_2_ in *oma1-1* under stress conditions suggests that the mechanism leading to enzymatic deficiency of these complexes is also connected with an assembly/stability defect. Furthermore, in contrast to the reduction in protein levels of complexes V, I, and supercomplex I + III_2_ in *oma1-1*, our analysis revealed that the complex IV accumulates in response to the lack of AtOMA1, independent of the plant growth conditions. The constancy in activity and abundance of complex IV was estimated by BN-PAGE (**Figures 4A,B**), increased protein amount of COX2 was observed by SDS-PAGE (**Figure 4C**), and the unchanged level of transcripts encoding core subunits of complex IV was detected by RT-PCR (Supplementary Figure S8), all these results indicating that the most likely effect of absence of AtOMA1 is the increased stability of complex IV. Based on our experiments, however, we cannot exclude the possibility that the enhanced abundance of complex IV in *oma1-1* is due to an increase in efficiency of translation of its subunits. The control of assembly/stability of OXPHOS complexes by AtOMA1 most probably involve a proteolytic function since no chaperone-like domain has been identified in OMA1. It is, thus, plausible that this proteolytic control is associated with factor(s) that regulate formation/stabilization of the OXPHOS complexes.

Impairment of mitochondrial bioenergetic function caused by the loss of OMA1 was also found in yeast and metazoans (Bohovych et al., 2015). In contrast to plants, however, the loss of Oma1 in these organisms affects the stability of RSCs without impeding individual electron transfer chain units. In yeast, the Oma1 protease controls the stability of supercomplexes III/IV, whereas in mouse fibroblasts, it stabilizes supercomplexes I/III/IV (Bohovych et al., 2015). The role of this protease in plants also appears to be connected with the fine-tuning of respiratory function, but not through the control of the complex IV-containing supercomplexes, which have not been identified in Arabidopsis mitochondria (Dudkina et al., 2006). Identification of the molecular bases of differences between plants and other eukaryotes requires further investigations. Altogether, these findings establish that the OMA1 protease is an important conserved factor that contributes to the functionality of the electron transfer chain in yeast and higher animals and to the OXPHOS system in plants.

A well-recognized proteolytic substrate of OMA1 in mammalian mitochondria is Yme1L protease, an ATP-dependent metalloprotease anchored to the inner membrane (Stiburek et al., 2012; Rainbolt et al., 2015). The western blot results obtained in this study show a substantial increase in the abundance of FTSH4 protease, a plant homolog of Yme1L, at the protein level in *oma1-1* (**Figure 6B**), suggesting that this protein could be under the proteolytic control of AtOMA1, as in mammals. A second, but not mutually exclusive explanation is based on the findings that both Arabidopsis FTSH4 (Kolodziejczak et al., 2007) and AtOMA1 (the present study) have been implicated in the control of complex V assembly/stability. In this case, the increased amount of FTSH4 could be a compensatory response to the lack of AtOMA1. Leaving aside the reason for accumulation of FTSH4 in *oma1-1*, it should be emphasized that this accumulation occurs under optimal conditions, whereas in mammals, the Yme1L degradation is stress-induced. Further studies are required to understand the relationship between FTSH4 and OMA1 proteases in plant mitochondria.

## Conclusion

Our study shows that in contrast to yeast, the absence of a single mitochondrial ATP-independent protease, with the exception of AtOMA1, is not critical for mitochondrial bioenergetic function as well as overall development and morphology in Arabidopsis. Our investigations highlight the importance of AtOMA1 in maintaining the functionality of the mitochondrial OXPHOS system. Its bioenergetic role was proven by the level of activity/abundance of the OXPHOS complexes as well as by the respiration rate, maximal OXPHOS capacity, and impaired ATP production. Further investigations are needed to gain a detailed understanding of the association of AtOMA1 protease with the functionality of the OXPHOS system in plant mitochondria.

## Accession Numbers

The sequences of mammalian MmIMP1 and MmIMP2 used in Supplementary Figure S1 are available in the GenBank under accession number Q96LU5 and Q96T52, respectively. The GenBank accession numbers of other gene and protein sequences are listed in Supplementary Tables S2 and S6.

## Author Contributions

HJ and IM designed the research. IM, RS-B, MH-C, MK, and AG performed the experiments. HJ, IM, RS-B, MH-C, and MK analyzed the data. HJ wrote the article with input of IM, RS-B, MH-C, and MK. All authors read and approved the final manuscript.

## Conflict of Interest Statement

The authors declare that the research was conducted in the absence of any commercial or financial relationships that could be construed as a potential conflict of interest.

## Footnotes

1 http://imagej.nih.gov/ij/

2 www.euroscarf.de

3 http://octopus.cbr.su.se/
