# Supplementary material for: AtOMA1 Affects the OXPHOS System and Plant Growth in Contrast to Other Newly Identified ATP-Independent Proteases in Arabidopsis Mitochondria
Source: Front Plant Sci. 2017 Sep 7;8:1543. doi: 10.3389/fpls.2017.01543 (PMC5594102; doi:10.3389/fpls.2017.01543)
Supplement: Supplementary file 1 [file Table_1.docx]

**Table S1.** Hydrophobic regions in the protein sequence of Arabidopsis ATP-independent proteases predicted by the OCTOPUS (http://octopus.cbr.su.se/).

| ***A. thaliana* ATP-independent proteases** | | **Number of hydrophobic regions**  **according to the OCTOPUS software** |
| --- | --- | --- |
| **Name** | **Locus** |  |
| AtIMP1a | At1g53530 | 1 |
| AtIMP2 | At2g31140 | non detected/available |
| AtATP23 | At3g03420 | non detected/available |
| AtOMA1 | At5g51740 | 2 |
| AtOCT1 | At5g51540 | 1 |
| AtICP55 | At1g09300.1 | non detected/available |

**Table S2.** Primers used for plasmid construction, cloning and genotyping.

| **Gene name** | **GenBank Accession Number** | **Locus** | | **Forward primer** | | **Reverse primer** |
| --- | --- | --- | --- | --- | --- | --- |
| **Plasmid construction and cloning** | | | | | | |
| *ScOCT1* | 853724 | YKL134C | | CACCATGCTTCGCACGATAATATTG | | AGACTTGTGAGACTGTGCTATAAAT |
| *ScIMP1* | 855182 | YMR150C | | CACCATGACGGTTGGTACACTTCCC | | GTTGCTCTTAGCCTGCACATCTAGA |
| *ScIMP2* | 855051 | YMR035W | | CACCATGTTTCGAGCCGGATCATCA | | TTCTAAAATGGCTCTTTTGCTAATG |
| *ScICP55* | 856811 | YER078C | | CACCATGTTACATAGGATAAACCCA | | CAGTGGTGCCACTTGGTCCTCCTCG |
| *ScOMA1* | 115209 | YKR087C | | CACCATGTTACGCAACATCATCAGG | | CATTGAGAAAAAACTTTTATAATAG |
| *OCT1* | 835228 | AT5G51540 | | CACCATGTGGAAGCTGACGCGGCGA | | TAGTCTAAGCTCGTTTAAAAGATAC |
| *IMP1a* | 841788 | AT1G53530 | | CACCATGAGAATGACATTTTTGAGT | | TCTCAATGACCCAAAGTATTC |
| *IMP1b* | 2745760 | AT1G23465 | | CACCATGACGTCGCCGTCATCGTCT | | TACCCTCCATAGCACTCTGCC |
| *IMP2* | 817670 | AT2G31140 | | CACCATGGCTTCAATCTCGACTTGG | | TGCCTTATGGTTCTTAGCCAT |
| *ICP55-1* | 837451 | AT1G093000.1 | | CACCATGCAGTTTCTTGCGCGGAAT | | ACCTTGCAAAGAGAAACTGGC |
| *ICP55-2* | 837451 | AT1G09300.2 | | CACCATGCTCAGATGGTTCGCTCTT | | ACCTTGCAAAGAGAAACTGGC |
| *OMA1* | 835248 | AT5G51740 | | CACCATGTCATGGTACAGAAGAACA | | AAGAAAGCCTTCAACGCCAGT |
| *ATP23* | 821258 | AT3G03420 | | CACCATGGAAGATGCAGCTGCT | | AGGAGCTCTATCAAAAGGCTT |
| *ATP23FLAG* | 821258 | At3g03420FLAG | | CACCATGGAAGATGCAGCTGCT | | TCACTTATCGTCGTCATCCTTGTAATCCTTATCGTCGTCATCCTTGTAATCCTTATCGTCGTCATCCTTGTAATCAGGAGCTCTATCAAAAGGCTT |
| *pTZCYTB2* | - | - | | TCCGAGCTCATGCTAAAATACAAACCTTTA | | TTCATCTAGAGTTGTCTATTTGGCCATTATG |
| CAMV 35S | AJ007626.1 | - | | ACGTAAGGGATGACGCACAATCCCAC | | - |
| **Genotyping (selection of T-DNA insertion lines)** | | | | | | |
| **Plant line** | **NASC number** | | **Gene name** | | **LP or LB primer** | **RP primer** |
| *icp55-1* | GABI_893A04 | | AT1G093000.1 | | CATTCGCTGAGCACCTCTTAC | AAGTGATGAACGCGGTTTATG |
| *icp55-2* | WiscDsLox507A03 | | AT1G093000.1 | | AAACGACGTGATCGATTGTTC | ATCAAATGACTGGCTTTGGTG |
| *atp23-1* | SAIL_135_G10 | | AT3G03420 | | ACTGACGTCACCTGTGCCTAC | TCTCTTGAAATGGCAATCACC |
| *atp23-2* | SALK_093517C | | AT3G03420 | | CTTCTCGAAGACACGTTTTGC | TGATTCCAAAACCCTAAACCC |
| *oct1-1* | SALK_077448 | | AT5G51540 | | TAAGTTCTCGTTTCAAACGCC | TGCTGAACATAAACAACCCAG |
| *imp1-1* | SALK_094274 | | AT1G53530 | | TTGAAAAATCAAGGGATCTTCG | TGACGAGTTGGGATGGTCTAG |
| *oma1-1* | SALK_088054C | | AT5G51740 | | AGCTATTGGAACGAAAGCATG | TTTTCACCATTGATTGGTTCC |
| *oma1-2* | SALK_012114C | | AT5G51740 | | TATCAATCCAAACCCGTTCTG | GATCGAGGTAGAATCTTCGGG |
| *imp2-1* | SALK_080262 | | AT2G31140 | | GGCCCACTTCTAGTTCCGTAC | ACACAATAAACGCAGATTCCC |
| *imp2-2* | SALK_080280 | | AT2G31140 | | ATAGCTAGAAGCCTACAATACTTTCC | CACAACATCAACATTGTCTTGTG |
| *imp2-3* | SALK_080264 | | AT2G31140 | | GGCCCACTTCTAGTTCCGTAC | GATTCCCCTAAACCGGAATTC |
| *imp2-4* | SALK_080272 | | AT2G31140 | | TCGTTTAGGTGCTTCCTTTTTC | AGTCATCATGAGACATTGAGCAC |
| LB-SALK | - | | - | | ATTTTGCCGATTTCGGAAC | - |
| LB-SAIL | - | | - | | TAGCATCTGAATTTCATAACCAATCTCGATACAC | - |
| LB-GABI | - | | - | | ATATTGACCATCATACTCATTGC | - |
| LB-WiscDsLox | - | | - | | AACGTCCGCAATGTGTTATTAAGTTG | - |

**Table S3.** Primers used for yeast strain construction.

| **Primer Name** | **Forward primer** | **Reverse primer** |
| --- | --- | --- |
| Coa2 | GTAACCGATGCATGGGTTTT | AGGAAGCGAATAAGAATAGCG |
| delCoa2 | AGAGCAGTCACTAGAAACAAAATAGTCAATAACCTATACTCGGATCCCCGGGTTAATTAA | TTAAATCTTTTTACTTAAAGCATCCTTCCTCTTTAACTCC GAATTCGAGCTCGTTTAAAC |

**Table S4.** pENTR and pDEST vectors used in this study.

| **Vector and reference** | **Vector description** | **Construct** |
| --- | --- | --- |
| pENTR (Invitrogen) | Donor vector | all entry clones |
| p2GWF7  (Joubes et al., 2004) | Gateway destination vector for fusing GFP tag to C-terminal of the gene and transient expression in protoplasts | AtOCT1-GFP,  AtOMA1-GFP,  AtIMP1a –GFP,  AtIMP1b-GFP,  AtIMP2-GFP,  AtATP23-GFP |
| pGWB514  (Nakagawa et al., 2007) | Gateway destination vector for stable transformation | AtATP2-FLAG |
| pGWB551  (Nakagawa et al., 2007) | Gateway destination vector for fusing GFP tag to C-terminal and stable transformation | AtIMP1a –GFP,  AtIMP1b-GFP,  AtIMP2-GFP, |
| pGWB511  (Nakagawa et al. 2007) | Gateway destination vector for fusing FLAG tag to C-terminal and stable transformation | AtOMA1-FLAG |
| pVV209  (Mullem V Van et al., 2003) | Gateway destination vectors for fusing HA tag to C-terminal of the gene and expression in yeast | AtOCT1-HA,  AtOMA1-HA,  AtIMP1a –HA,  AtIMP2-HA,  AtICP55.1-HA,  AtATP23-HA,  OMA1-HA  IMP1-HA  IMP2-HA  ATP23-HA |

**Table S5.**

List of antibodies used in this study.

| **Antibody** | **Antibody Type** | **Source (cat. no.) & References** |
| --- | --- | --- |
| FTSH10 | polyclonal | Agrisera (AS07 251), Piechota et al., 2010 |
| FTSH3 | polyclonal | Agrisera (AS07 204), Piechota et al., 2010 |
| FTSH4 | polyclonal | Agrisera (AS07 205), Gibala et al., 2009 |
| PREP | polyclonal | kind gift from Prof. Elzbieta Glaser, University of Stockholm, Sweden |
| OOP | polyclonal | kind gift from Prof. Elzbieta Glaser, University of Stockholm, Sweden |
| RBL12 | polyclonal | Antiserum was produced in rabbits against a synthetic peptide corresponding to residues 239 to 252 of AtRBL12 sequence  (GenScript, USA) |
| CYTC | polyclonal | kind gift from Prof. Philippe Giegé,  CNRS, Strasbourg, France |
| LHCB2 | polyclonal | Agrisera (AS01 003) |
| VDAC1-5 | polyclonal | Agrisera (AS07 212) |
| GFP | polyclonal | Thermo Fisher Scientific (11814460001) |
| Atp6 (*S. cerevisiae*) | polyclonal | kind gift from Marie-France Giraud, University of Bordeaux, France |
| FLAG | monoclonal | Thermo Fisher Scientific (F1804) |
| HA | monoclonal | Thermo Fisher Scientific (11867423001) |
| NAD6 | polyclonal | Agrisera (AS15 2926) |
| NAD9 | polyclonal | kind gift from Dr. Jose M. Gualberto, CNRS, Strasbourg, France |
| ATP2 | polyclonal | Agrisera (AS05 085) |
| ATP1 | monoclonal | Abcam (ab14748) |
| COX2 | polyclonal | Agrisera (AS04 053A) |

**Table S6.** Primers used for qRT-PCR.

| **Gene name** | **GenBank Accession Number** | **Locus** | **Forward primer** | **Reverse primer** |
| --- | --- | --- | --- | --- |
| **ATP-dependent proteases** | | | | |
| *MPPα* | 820896 | AT3G16480 | CATTCATTCTGCTGGTTA | TTAGGAAGGTCAGAAGTTA |
| *LON1* | 832744 | AT5G26860 | GTAGGCAAAACTAGTATT | TATCAATCTCATCGATTAG |
| *LON4* | 819748 | AT3G05790 | TAGGTAAAACTAGCATTG | TATCAATCTCATCGATCA |
| *CLPX* | 835416 | AT5G53350 | TCCGTGGTAAAGGAGCATTGGC | ATCCCTCCTTTGTCGTCTGTGG |
| *FTSH3* | 817456 | AT2G29080 | CGCCGAGCTTTTACTAGAGAAA | AGCTCCATCATCCACCACAGGC |
| *FTSH4* | 817154 | AT2G26140 | TCAGCATGCCGCGGTGAATATG | TGCTTCGGGCGAAACCAATTCC |
| *FTSH10* | 837265 | AT1G07510 | CGCTGAGCTCTTGCTAGAGAAG | ATTCCATCATCCTCCACAGGCT |
| **ATP-independent proteases** | | | | |
| *ATP23* | 821258 | AT3G03420 | CATGATTCGTAGGAGTTTCC | TGCAGACAGTTATCCCTCTT |
| *OMA1* | 835248 | AT5G51740 | ACTGTGATTGGGCATGAGGTTGG | AGGGCGGACATAGTGTTCACAAG |
| *OCT1* | 835228 | AT5G51540 | ATGCTCTTCACTCTCTGCTC | CTTCTCGGGTATTGTCTCAC |
| *IMP1a* | 841788 | AT1G53530 | ATCAATCGTCGCCAAGTTTC | TACAACGTCACCGAGTCCAA |
| *ICP55* | 837451 | AT1G09300.1 | GCTTGGGAAGGAGAAGTTG | CGGGTAATTTGCTTATCGG |
| *RBL12* | 838441 | AT1G18600 | TTGTTCTTGGGTTAGTTATAG | TGTCCAATATCAATATGACTG |
| *OOP* | 836688 | AT5G65620 | TATGAAAATGGCTACTGTTG | TGAGTCATACTGTCAGATTC |
| *PREP2* | 841387 | AT1G49630 | AGCCATTAAAGTATGAGGAA | TATAGTAACACAATGAGGGT |
| *ACT2* | 821411 | AT3G18780 | ATCGAGAAGAACTATGAATTAC | AAGTGCTGTGATTTCTTT |
| **Complex I** | | | | |
| *nad2* | 3371312 | ATMG00285 | TTTACATATTTCTGCCAA | TAAAAACATAAACTTTGAGG |
| *nad6* | 814580 | ATMG00270 | TATGATGTTCCATATTCAA | GACAGAATAGTAGGTATAA |
| *CA2* | 831326 | AT5G14740 | CCGTCGAACGAATTAAGGAAGGC | ACCATACAAAGCAGGATTGGTCTC |
| **Complex IV** | | | | |
| *cox1* | 814620 | ATMG01360 | GATCGAAACTTTAATACAAC | AACAAGAAATCCTAAGACA |
| *cox2* | 814573 | ATMG00160 | CATCACATTTGACACCTG | CAGCCATTACTATCAAAGC |
| *COXVB* | 844363 | AT1G80230 | CTCCATATTCTCTCGTTTA | CAACTATTCTCATGTCGTA |
| **Complex V** | | | | |
| *atp6* | 814588 | ATMG00410 | AGGATTGTCCGGGCCTTAATCC | ATGCCTCACACTTCTCGCGTATC |
| *atp9* | 814613 | ATMG01080 | TGTCCAAGATCAAAGGAA | CAGAATGAATCAAAGAACT |
| *ATP2-1* | 830768 | AT5G08670 | TAGTGTACGGACAAATGA | GTAGTAATTCGCTCTTGA |
| *ATP2-3* | 830770 | AT5G08690 | AATGACTTGTACAGAGAA | AATGTTGTCAATGAAAAG |
