# Supplementary material for: AtOMA1 Affects the OXPHOS System and Plant Growth in Contrast to Other Newly Identified ATP-Independent Proteases in Arabidopsis Mitochondria
Source: Front Plant Sci. 2017 Sep 7;8:1543. doi: 10.3389/fpls.2017.01543 (PMC5594102; doi:10.3389/fpls.2017.01543)
Supplement: Supplementary file 2 [file Image_1.pdf]

```
                     *        20         *        4
MmImp1     : -------------------MLRGVLGKAFRLAGYTIQYG :  20
Imp1       : -------------------MTVGTLPIWSKTFSYAIRSL :  20
At1g53530  : ---------MRMTFLSYLKQWRGTAKEAFENVSIVAKFL :  30
At1g23465  : -----------MTSPS-SSFWNTASREAMKSGMFVAKVY :  27
MmImp2     : ----------------MAQSQSWARRCFKAFCKGFFVAV :  23
Imp2       : ----------------MFRAGSSKR--FLRNTLIAISWV :  21
At2g31140  : MASISTWFRYMAHKLEYSLTLSLKSHRSNKLSDRELIQI :  39


             0         *        60         *
MmImp1     : CIAHCAFEYVGGVVMCSGPSMEPTIQNS-DIVFAENLSR :  58
Imp1       : CFLHIIHMYAYEFTETRGESMLPTLSATNDYVHVLKNFQ :  59
At1g53530  : CLLHVTDRYIISTTHVHGPSMLPTLNLTGDVILAEHLSH :  69
At1g23465  : CFLHVTTNYLGFMAYAYGPSMIPTLHPSGNMLLAERISK :  66
MmImp2     : PVAVTFLDRVACVARVEGSSMQPSLNPG-GSQSSDVVLL :  61
Imp2       : PVLLTINNNVVHIAQVKGTSMQPTLNPQTETLATDWVLL :  60
At2g31140  : ICKNLFYGKITYLHSDKGPEMSPTMTANENTLLIRKIPI :  78


             80         *       100         *
MmImp1     : HFYGIQR------GDIVIAKSPSDPKSNICKRVIGLEGD :  91
Imp1       : NGRGIKM------GDCIVALKPTDPNHRICKRVTGMPGD :  92
At1g53530  : RFGKIGL------GDVVLVRSPRDPKRMVTKRILGLEGD : 102
At1g23465  : RYQKPSR------GDIVVIRSPENPNKTPIKRVVGVEGD :  99
MmImp2     : NHWKVRN-FEVQRGDIVSLVSPKNPEQKIIKRVIALEGD :  99
Imp2       : WKFGVKNPSNLSRDDIILFKAPTNPRKVYCKRVKGLPFD :  99
At2g31140  : ANTRFVF-----IGDAVVLKDPNDSDKYLVRRLAAVEGF : 112


             120         *       140         *
MmImp1     : KILSTS------------PSDVFKSRSYVPTGHVWLEGD : 118
Imp1       : LVLVDPSTIVNYVGDVLVDEERFGTYIKVPEGHVWVTGD : 131
At1g53530  : RLTFSADP----------LVGDASVSVLVPKGHVWIQGD : 131
At1g23465  : CISFVIDP----------VKSDESQTIVVPKGHVFVQGD : 128
MmImp2     : IVRTIG---------------HKNRLVKVPRGHMWVEGD : 123
Imp2       : TIDTKFP--------------YPKPQVNLPRGHIWVEGD : 124
At2g31140  : EMVSGD---------------EKEEPFVLEKNQCWVTAE : 136


              160         *       180         *
MmImp1     : N----LQNSTDSRYYGPIPYGLIRGRIFFK--------- : 144
Imp1       : N----LSHSLDSRTYNALPMGLIMGKIVAANNFDKPFWD : 166
At1g53530  : N----LYASTDSRHFGPVPYSLIEGKALLR--------- : 157
At1g23465  : Y----THNSRDSRNFGPVPYGLIQGRVLWR--------- : 154
MmImp2     : H----HGHSFDSNSFGPVSLGLLHAHATHI--------- : 149
Imp2       : N----YFHSIDSNTFGPISSGLVIGKAITI--------- : 150
At2g31140  : NQELKAKEAYDSRTFGPVSTADIVGRAIYC--------- : 166


               200         *       220         *
MmImp1     : --IWPFSDFGFLRDSPNGQRFSDD--------------- : 166
Imp1       : GSIRNIWGFKWINNTFLDVQAKSN--------------- : 190
At1g53530  : --VWPPEYFGSLR-------------------------- : 168
At1g23465  : --V------------------------------------ : 155
MmImp2     : --LWPPERWQRLESVLPPERCPLQTGEK----------- : 175
Imp2       : --VWPPSRWGTDLKLSTGRDCISKRAILE---------- : 177
At2g31140  : --LRTAVDHGPVRNSQTAMGQDSPILAVELDVDEMAKNH : 203


MmImp1     : -- :   -
Imp1       : -- :   -
At1g53530  : -- :   -
At1g23465  : -- :   -
MmImp2     : -- :   -
Imp2       : -- :   -
At2g31140  : KA : 205
```

Figure S1. Multiple sequence alignment of the Imp family of proteases. The amino acid sequences of Imp1 and Imp2 from *S. cerevisiae*, MmImp1 and MmImp2 from mammals and the Arabidopsis homologs (At1g53530, At1g23465, At2g31140) were aligned with ClustalW. The Imp1 (RX5P) and Imp2 (NX5S) - specific motifs are colored in green and yellow, respectively.

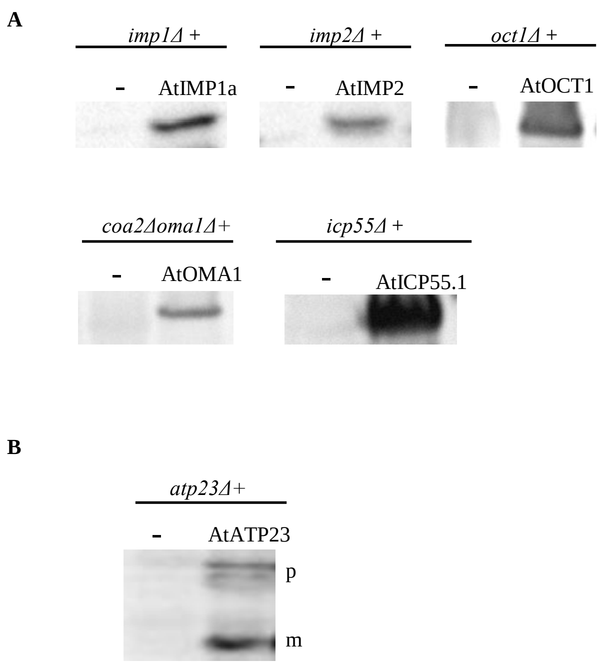

Figure S2. Immunodetection of plant proteases (AtIMP1a, AtIMP2, AtOCT1, AtOMA1, AtICP55.1 and AtATP23) in yeast mutants lacking the respective protease. Immunodetection was performed using anti-HA antibodies. **(A)** Isolated mitochondria from yeast mutant carrying HA-tagged version of plant protease. **(B)** Total protein extract from yeast mutant expressing AtATP23 fused with a mitochondrial targeting sequence derived from the yeast cytochrome b2 protein. p-precursor protein, m-mature protein, "-" untransformed yeast mutant cells.

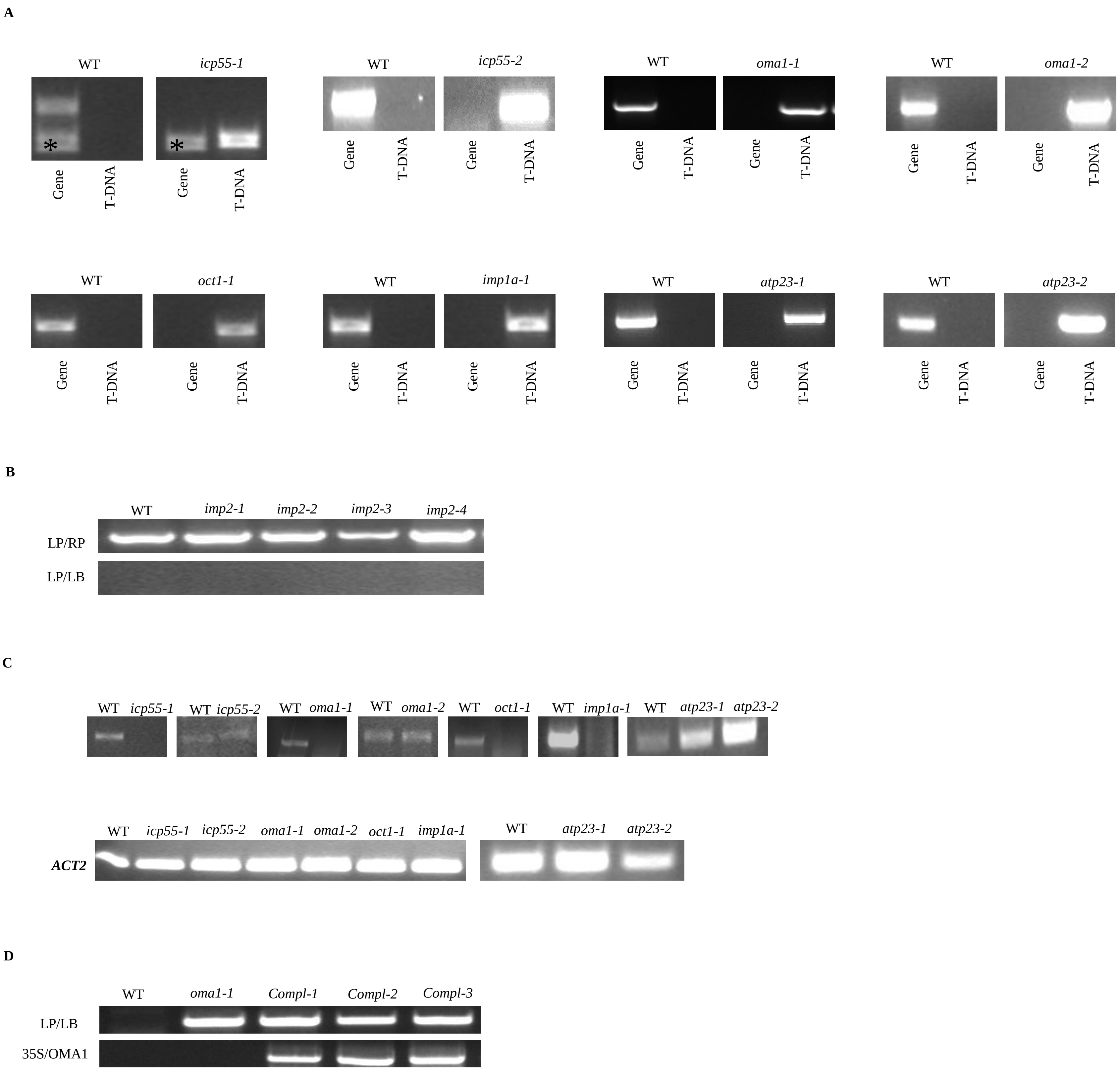

Figure S3. Characterization of T-DNA insertional mutants used in this work and AtOMA1- Complemented lines. **(A)** Homozygous T-DNA insertion plants were identified by PCR using the respective gene specific primer pair LP and RP (named as Gene) (Table S2) as well as the T-DNA specific primer pair LP and/or RP and LB (named as T-DNA) (Table S2); *- unspecific band. **(B)** Lack of T-DNA verified with primers specific for T-DNA insertion (LP/LB) in *imp2* lines: SALK_080262 (*imp2-1*), SALK_080280 (*imp2-2*), SALK_080264 (*imp2-3*), SALK_080272 (*imp2-4*). **(C)** Analysis of expression of respective protease by RT-PCR in wild-type (WT) and homozygous mutant lines. Amplification of the transcript for the *ACT2* gene was used as a control. **(D)** Confirmation of three independent AtOMA1-Complemented lines (*Compl-1*, *Compl-2*, *Compl-3*) by PCR. The upper reaction was performed using primers specific to T-DNA insertion (LP/LB), lower reaction was performed using primers specific for CaMV35S promoter and *OMA1* cDNA (35S/OMA1) (Table S2).

Gene	Salk line (NASC line)	T-DNA inserted in	Transcript	Plant line	Gene model and T-DNA line
AtICP55 (At1g09300)	GABI_0893A04 (N363490)	exon	-	*icp55-1*	
AtICP55 (At1g09300)	WiscDsLox507A03 (N859299)	3'-UTR	+	*icp55-2*	
AtOMA1 (At5g51740)	Salk_088054C (N654767)	exon	-	*oma1-1*	
AtOMA1 (At5g51740)	Salk_012114C (N661499)	promoter	+	*oma1-2*	
AtOCT1 (At5g51540)	Salk_077448 (N577448)	exon	-	*oct1-1*	
AtIMP1a (At1g53530)	Salk_094274 (N682671)	exon	-	*imp1a-1*	
AtATP23 (At3g03420)	Sail_135_G10 (N806583)	promoter	+	*atp23-1*	
AtATP23 (At3g03420)	Salk_093517C (N663359)	5'-UTR	+	*atp23-2*	

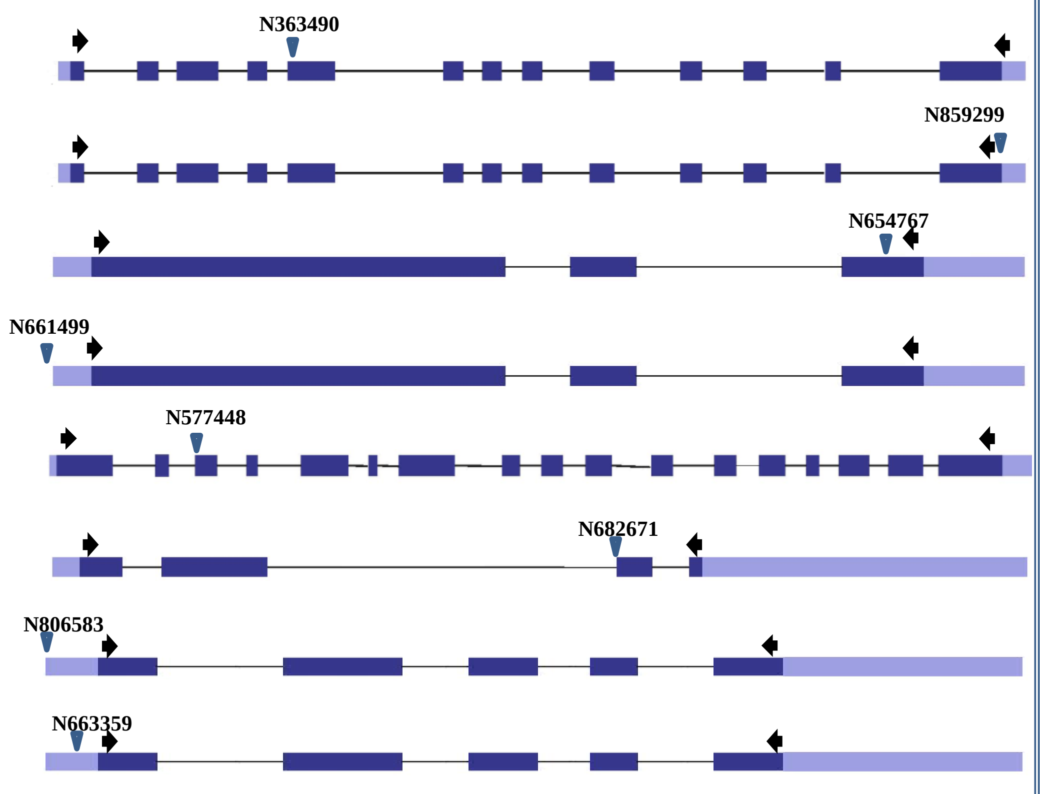

Figure S4. Schematic illustration of the T-DNA insertions within genes coding Arabidopsis ATP-independet proteases with respect to DNA sequence and intron/exon regions. Exons are presented by navy blue box, and introns by lines. T-DNA insertion, verified by sequencing, is shown as a triangle with NASC line name above. "+" and "-" indicate the presence or absence of a full length transcript in the mutant, respectively. The positions of primers used for transcript verification are indicated by black arrows.

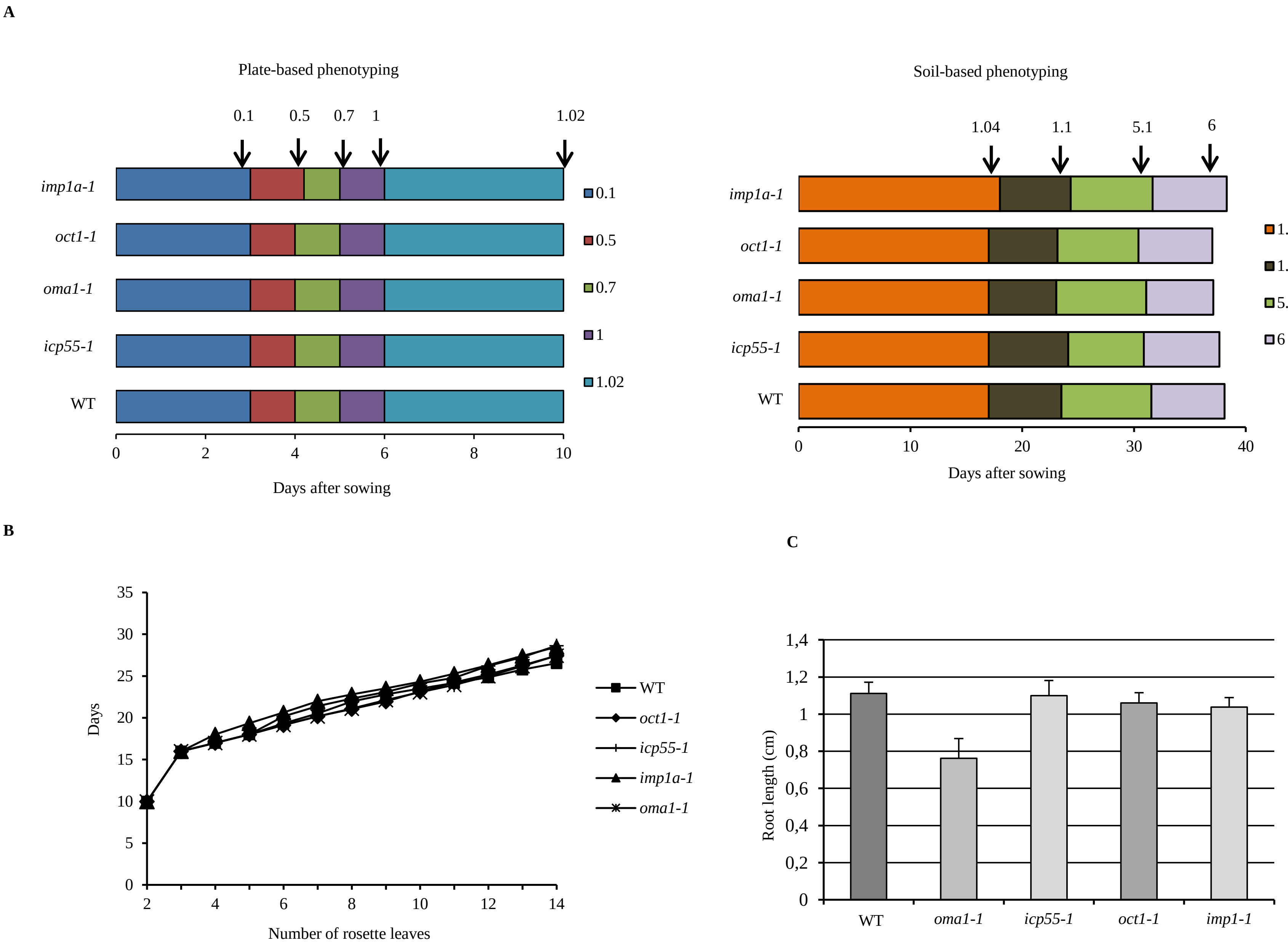

Figure S5. Phenotypic analysis of wild-type and T-DNA insertion lines under LD, 22°C condition. **(A)** Growth stage progression analysis for the plate- and soil-grown plants. The end of growth stages was determined according to Boyes *et al*., (2001). **(B)** Number of rosette leaves >1 mm long produced over time. (**C)** The length of the roots of 5-day-old seedlings. Mean values ± SD from at least ten individual plants.

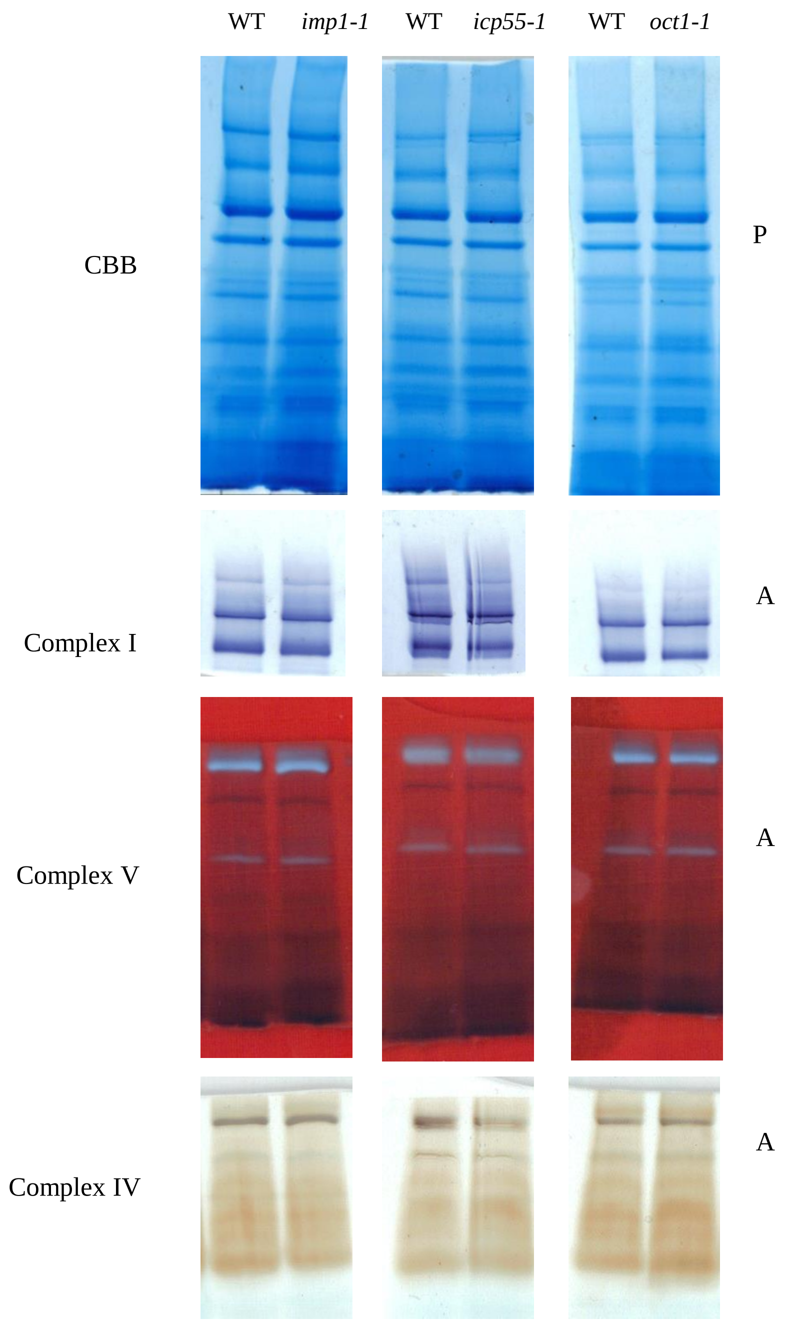

Figure S6. In-gel enzyme activity (A) and Coomassie blue (P) staining of mitochondrial OXPHOS complexes separated by blue-native polyacrylamide gel electrophoresis. Mitochondria for BN-PAGE were isolated from 14-day-old plants grown under optimal conditions (LD, 22°C).

A

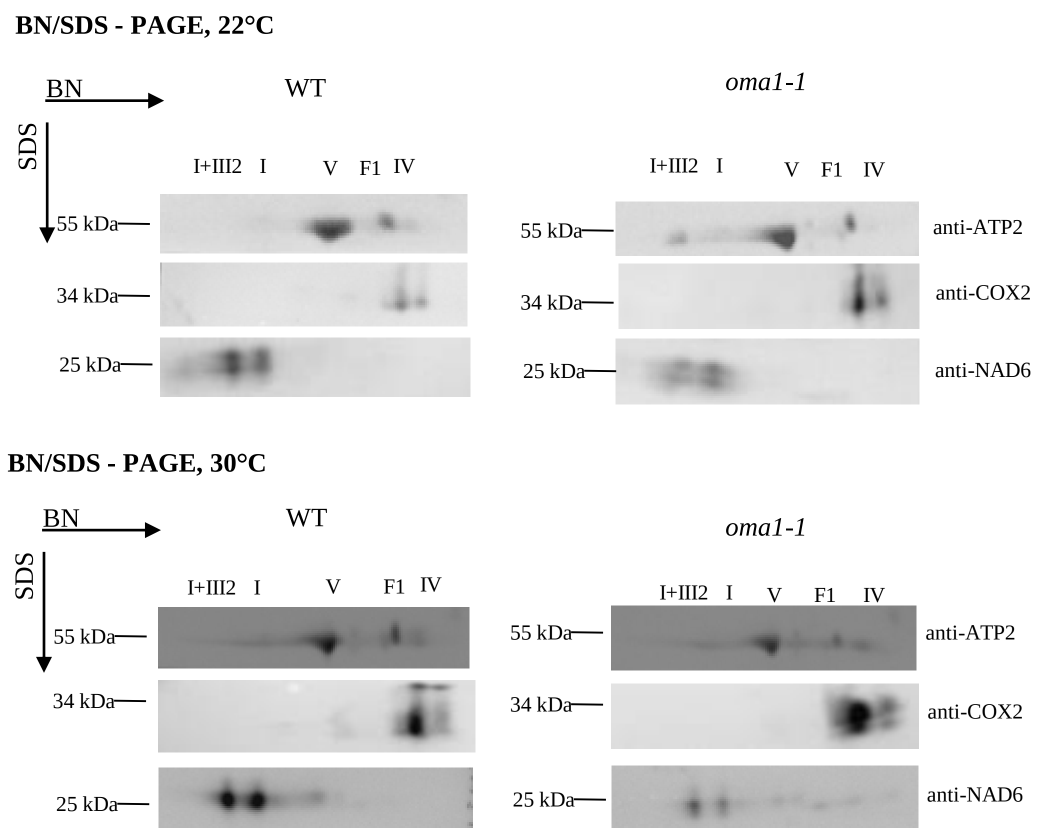

Figure S7. BN/SDS-PAGE electrophoresis followed by immunoblotting with antibodies directed against subunits of complexes I, IV and V. Mitochondria were isolated from 14-day-old WT and *oma1-1* plants grown under LD, 22°C and 30°C.

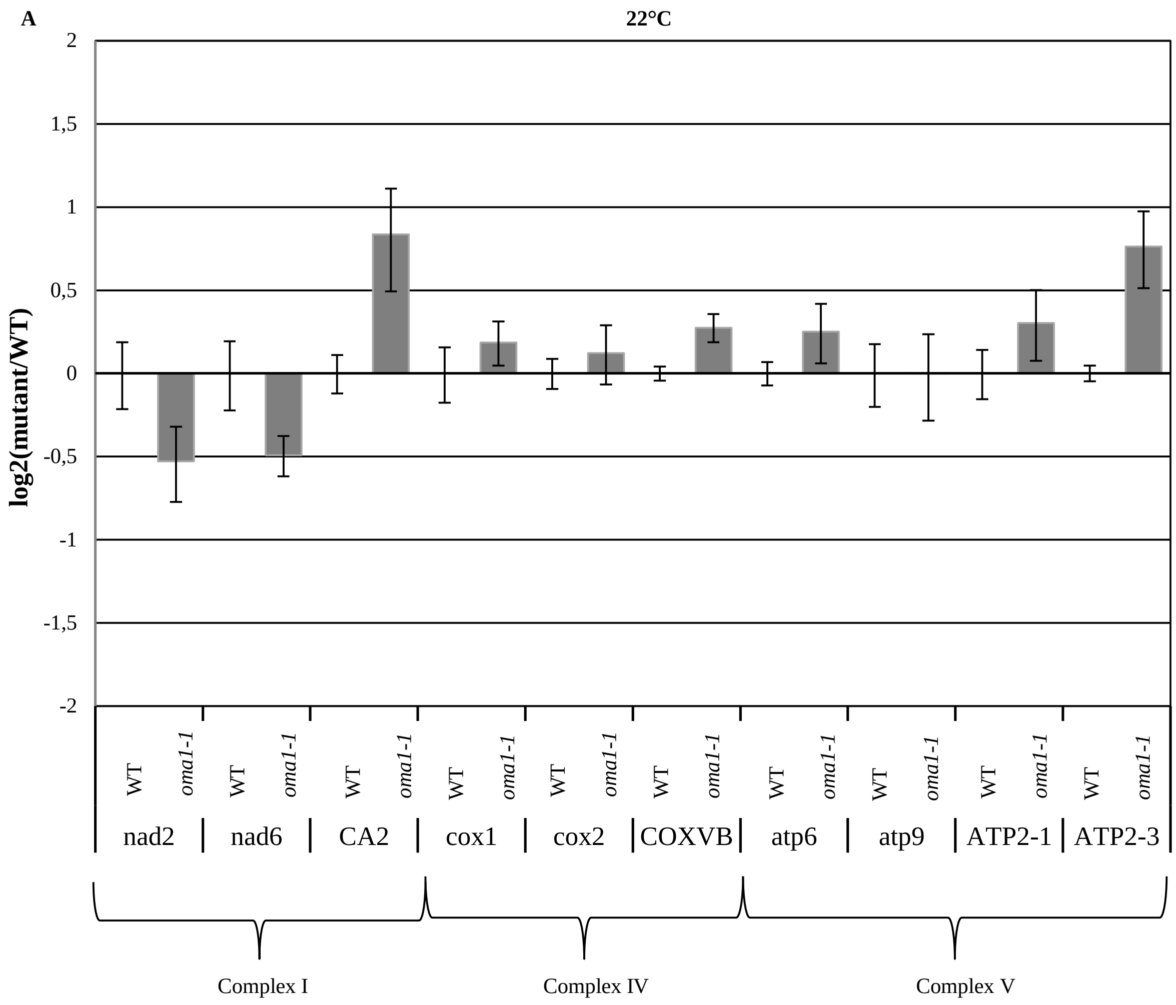

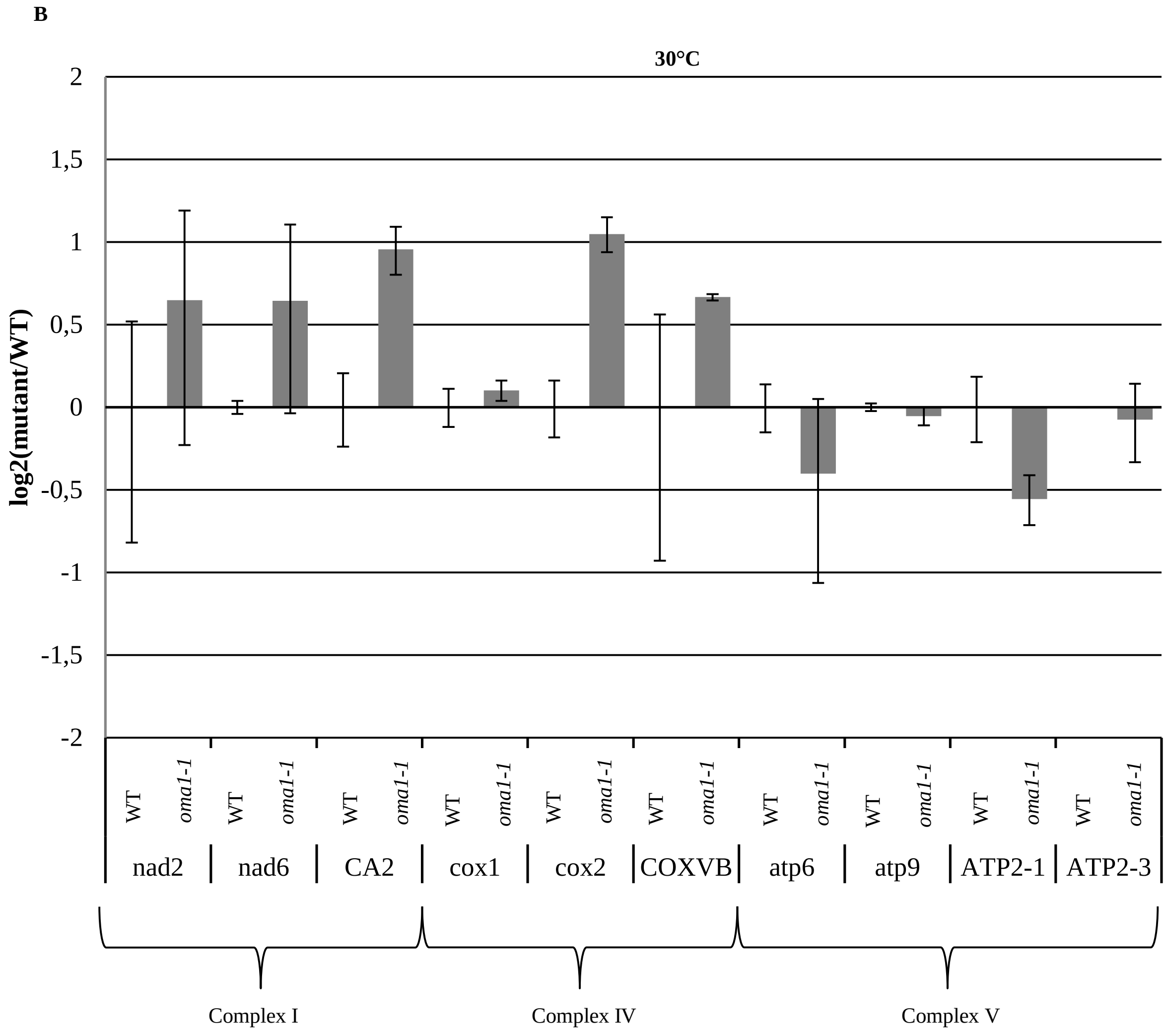

Figure S8. The transcript level of the selected genes coding for subunits of the OXPHOS complexes in *oma1-1* mutants grown under LD, 22°C **(A)** and LD 30°C **(B)** compared to the wild-type plants. Analysis was performed by quantitative real-time PCR. Relative abundance of transcripts is expressed as log2 ratio. Mean values ± SD from three independent experiments are shown.
